# The complex hexaploid oil‐Camellia genome traces back its phylogenomic history and multi‐omics analysis of Camellia oil biosynthesis

**DOI:** 10.1111/pbi.14412

**Published:** 2024-06-24

**Authors:** Huaguo Zhu, Fuqiu Wang, Zhongping Xu, Guanying Wang, Lisong Hu, Junyong Cheng, Xianhong Ge, Jinxuan Liu, Wei Chen, Qiang Li, Fei Xue, Feng Liu, Wenying Li, Lan Wu, Xinqi Cheng, Xinxin Tang, Chaochen Yang, Keith Lindsey, Xianlong Zhang, Fang Ding, Haiyan Hu, Xiaoming Hu, Shuangxia Jin

**Affiliations:** ^1^ College of Biology and Agricultural Resources, Huanggang Normal University Huanggang Hubei China; ^2^ National Key Laboratory of Crop Genetic Improvement, Hubei Hongshan Laboratory Huazhong Agricultural University Wuhan Hubei China; ^3^ Spice and Beverage Research Institute, Chinese Academy of Tropical Agricultural Sciences Wanning Hainan China; ^4^ Hubei Academy of Forestry Wuhan Hubei China; ^5^ National Key Laboratory of Crop Genetic Improvement and National Center of Plant Gene Research (Wuhan) Huazhong Agricultural University Wuhan Hubei China; ^6^ College of Agriculture, Shihezi University Shihezi Xinjiang China; ^7^ Department of Biosciences Durham University Durham UK; ^8^ Hubei Key Laboratory of Plant Pathology, College of Plant Science and Technology Huazhong Agricultural University Wuhan Hubei China; ^9^ School of Breeding and Multiplication (Sanya Institute of Breeding and Multiplication) Hainan University Sanya Hainan China

**Keywords:** Oil‐Camellia (*Camellia oleifera*), hexaploid, chromosome‐scale genome assembly, whole‐genome duplication, camellia oil biosynthesis

## Abstract

Oil‐Camellia (*Camellia oleifera*), belonging to the Theaceae family Camellia, is an important woody edible oil tree species. The Camellia oil in its mature seed kernels, mainly consists of more than 90% unsaturated fatty acids, tea polyphenols, flavonoids, squalene and other active substances, which is one of the best quality edible vegetable oils in the world. However, genetic research and molecular breeding on oil‐Camellia are challenging due to its complex genetic background. Here, we successfully report a chromosome‐scale genome assembly for a hexaploid oil‐Camellia cultivar Changlin40. This assembly contains 8.80 Gb genomic sequences with scaffold N50 of 180.0 Mb and 45 pseudochromosomes comprising 15 homologous groups with three members each, which contain 135 868 genes with an average length of 3936 bp. Referring to the diploid genome, intragenomic and intergenomic comparisons of synteny indicate homologous chromosomal similarity and changes. Moreover, comparative and evolutionary analyses reveal three rounds of whole‐genome duplication (WGD) events, as well as the possible diversification of hexaploid Changlin40 with diploid occurred approximately 9.06 million years ago (MYA). Furthermore, through the combination of genomics, transcriptomics and metabolomics approaches, a complex regulatory network was constructed and allows to identify potential key structural genes (*SAD*, *FAD2* and *FAD3*) and transcription factors (AP2 and C2H2) that regulate the metabolism of Camellia oil, especially for unsaturated fatty acids biosynthesis. Overall, the genomic resource generated from this study has great potential to accelerate the research for the molecular biology and genetic improvement of hexaploid oil‐Camellia, as well as to understand polyploid genome evolution.

## Introduction

Oil‐Camellia, in a broad sense, refers to more than 60 shrubs of the genus Camellia (Theaceae) whose seed kernels produce high‐quality edible oils (Lin *et al*., 2022). It is a subtropical evergreen shrub or small tree with high nutritional and medicinal values and is mainly distributed in China, Philippines, India, Japan, Brazil, Thailand and South Korea (Wang *et al*., 2013; Yang *et al*., 2016). Together with olive, oil palm and coconut, it has emerged as one of four major woody plant oil species in the world (Yang *et al*., 2022). Oil‐Camellia also has a long cultivation history, spanning over 2300 years and has been cultivated extensively as an oil crop in China (Lin *et al*., 2019). Currently, China is planting oil‐Camellia trees in 17 tropical and subtropical provinces in the south of Qinling Mountains, covering a total area of 4.6 million hectares (Luan *et al*., 2020).

Genomic information plays a fundamental role in crop genetic improvement programmes, and large‐scale population genomics analyses provide accurate information for identifying genomic variations underlying the selection of desirable traits (Bevan *et al*., 2017; Wang *et al*., 2018). Recently, chromosome‐level diploid oil‐Camellia genomes (*Camellia oleifera* var. ‘Nanyongensis’ (CON), *Camellia chekiangoleosa* Hu. (CCH) and *Camellia lanceoleosa*, 2n = 2x = 30) have also been reported, and genome size of 2.95 Gb, 2.73 Gb and 3.00 Gb were obtained, respectively (Gong *et al*., 2022; Lin *et al*., 2022; Shen *et al*., 2022). However, *C. chekiangoleosa* belongs to the section *Camellia*, *C. lanceoleosa* and *C. oleifera* belong to the same Oleifera, Sect, but are evolutionarily distantly related (Cui *et al*., 2022). The diploid CON is considered to be the ancestor species of the hexaploid *C. oleifera*, and they differ morphologically (Lin *et al*., 2022). Usually, chromosome polyploidization leads to giant plant vegetative organs and larger fruits, which results in significantly improved yield, quality and resistance (Van de Peer *et al*., 2017). Therefore, dissecting the genome of hexaploid *C. oleifera* can greatly promote genetic improvement of important traits such as oil yield and quality, important active substance components, and provide theoretical basis for revealing the molecular mechanism of oil Camellia genetic evolution and molecular breeding.

*Camellia* is the largest genus in the family Theaceae and it is divided into 18 groups including more than 200 species based on Chang's classification system (Chang, 1981). Extensive hybridization and polyploidization have resulted in challenging for the study of taxonomic and phylogenetic aspects of *Camellia* plants. Studies have shown that intra‐ and interspecific variations in the genus *Camellia* are pronounced with interspecific variation being significantly greater than intraspecific variation. In addition, there is a trend towards increased genome size in the genus *Camellia*, possibly associated with frequent polyploidization events, and this genus has extensive phylogenetic diversity (Huang *et al*., 2013, 2014). Gene family analysis of different diploid *Camellia* species exhibited the lack of spectrum‐specific whole‐genome duplication (WGD) in the genus *Camellia*. Single‐copy homology phylogenetic analysis revealed that CON and *Camellia lanceoleosa* were isolated from *Camellia sinensis* by 17.30 and 6.00–7.00 million years ago (MYA), respectively (Gong *et al*., 2022; Lin *et al*., 2022). In contrast, Theaceae (*Camellia chekiangoleosa* and *Camellia sinensis*) were isolated from *Actinidia chinensis* at about 71.22 (49.22–93.81) MYA (Shen *et al*., 2022). The origin of the *Camellia* genus occurred at approximately 14.30 MYA, with the likely origination of tea plants (*Camellia* sect. *Thea*) estimated at around 6.67 MYA. Furthermore, the separation of *Camellia* sect. *Thea* and *Camellia* sect. *Oleifera* is believed to have occurred over a time span exceeding 5.88–6.58 MYA. *Camellia* plants underwent a WGD followed by a massive expansion of a family of transcription factors (TFs) associated with resistance and secondary metabolism (Wu *et al*., 2022b). However, polyploidization in Camellia plants remains largely unknown.

The tea oil, extracted from seed kernel of Oil‐Camellia, is rich in monounsaturated fatty acids (account for more than 80% of the total oil) and has been so‐called ‘eastern olive oil’ because of its medical and healthcare functions for human health (Lin *et al*., 2022; Yang *et al*., 2016). Comparative analysis with the genome of *Camellia sinensis* indicated that the high oleic acid and oil content may be mainly due to the extended expression of homomeric acetyl‐coenzyme A carboxylase (ACCase) and the seed‐biased expression of genes encoding heteromeric ACCase, diacylglycerol acyltransferase, glyceraldehyde‐3‐phosphate dehydrogenase and stearoyl‐ACP desaturase (Gong *et al*., 2022).

An accurate and comprehensive reference genome sequence is crucial in revealing the origin and evolutionary history of a species, as well as the genetic foundation that underlies its phenotypic variation. Despite the economic importance and evolutionary significance of hexaploid oil‐Camellia, its genome has not yet been deciphered, mainly due to its high ploidy level, high repetitiveness, high homology and large genome size. Recently, the assembly of three diploid *Camellia oleifera* (Gong *et al*., 2022; Lin *et al*., 2022; Shen *et al*., 2022) genomes and the utilization of chromosome conformation capture sequencing (such as Hi‐C) to resolve haplotypes (Zhang *et al*., 2021) and polyploids (Chen *et al*., 2020; Jin *et al*., 2023; Peng *et al*., 2022; Song *et al*., 2023; Zhang *et al*., 2018, 2019) assembly have provided valuable strategy to dissect the evolutionary history of the hexaploid oil‐Camellia. Here, we combined high‐quality long PacBio HiFi reads together with Hi‐C reads and presented a chromosome‐scale genome assembly of the hexaploid *Camellia oleifera* (2n = 6x = 90) cultivar ‘Changlin40’, which is well known for its high‐quality Camellia oil (Zeng and Endo, 2019). The availability of this valuable genetic information provides us with the opportunity to gain insights into the evolutionary processes of hexaploid *Camellia oleifera* and further demonstrates the feasibility of using this high‐quality reference genome in identifying genes that underlie important agronomic traits.

## Results

### Chromosome‐scale assembly of hexaploid oil‐Camellia genome

**Figure 1:**
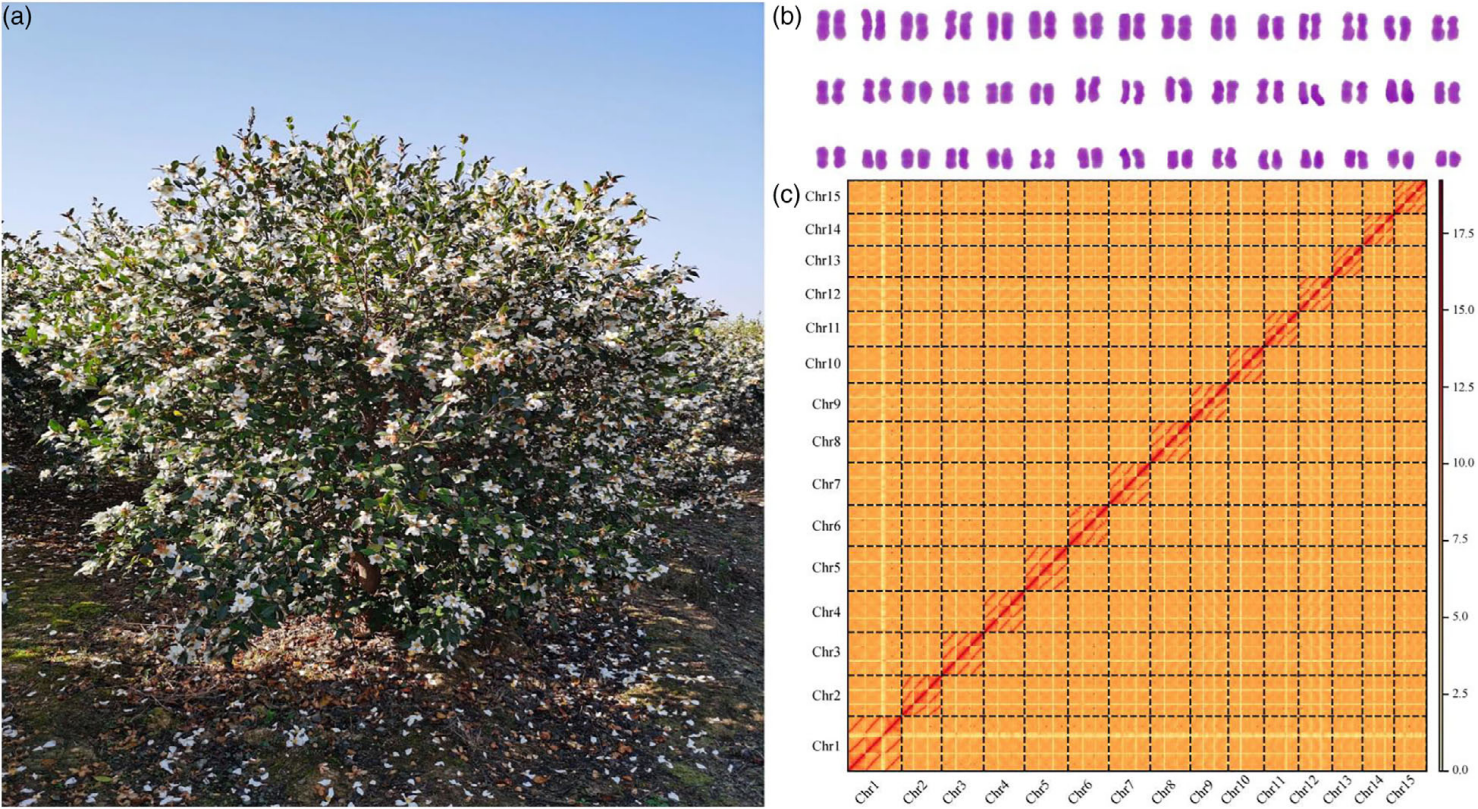
Genomic characteristics of the hexaploid Changlin40. (a) Image of Changlin40 in a field. (b) The karyotype of Changlin40 obtained via karyotype analysis. (c) Hi‐C heatmap showing the chromosomal interactions of intra‐ and interchromosomal within Changlin40. Each homoeologous chromosome group contains three pseudomolecules.

We selected a hexaploid (2n = 6x = 90) oil‐Camellia cultivar, Changlin40 (Figure 1a; Figure S1), to perform DNA sequencing and genome assembly. Changlin40, an exemplary cultivated species selected by the Subtropical Forestry Research Institute of the Chinese Academy of Forestry, stands as a prominent cultivar within the oil‐Camellia species. Renowned for its robust resistance and remarkable oil production capabilities. Notably, Changlin40 exhibits a substantial content of unsaturated fatty acids, specifically oleic acid and linoleic acid, which collectively account for approximately 90% of the total Camellia oil composition.

Based on k‐mer survey using 519.00 Gb Illumina reads data (Table S1), the Changlin40 genome size is estimated to be 8.80 Gb with 7.42% heterozygosity and repetitive sequences content of 81.00%, as well as have greater than 92.6% homozygous genome sequences (Figure S2). In addition, the karyotype analysis indicated Changlin40 was hexaploidy with 90 chromosomes (Figure 1b).

Before genome assembly, we attempted to determine whether hexaploid Changlin40 belongs to allo‐ or auto‐polyploidy species through cytological observation. Cytological observations revealed that the meiosis of pollen mother cells in Changlin40 is normal (Figure S3). The process of chromosome pairing occurs at zygotene, resulting in the formation of 45 bivalents at diakinesis and the presence of multivalents and univalents is not observed. Homologous chromosomes can separate without any aberrant phenomena at anaphase I, such as chromosome laggards and bridges. In anaphase II and telophase II, the sister chromatids are typically separated into two distinct poles. During the tetrad stage, four distinct daughter cells are produced from the pollen mother cells after two consecutive divisions (Figure S3). No instances of multiple divisions or micronuclei were observed. Therefore, the chromosome behaviour of Changlin40 in meiosis is indistinguishable from that of diploid organisms.

To overcome the challenge of highly complicated polyploid genome assembly, a total of 379.58 Gb (~42x, subread N50 = 15 533 bp) of high‐quality long PacBio HiFi reads (Table S2) and 789.10 Gb (~84x) Hi‐C reads (Table S3) were obtained and combined to generate the preliminary assembly using the hifiasm program with ‘‐hic’ mode, resulting in an initial assembly consisted of 59 032 contigs with the total assembly size of 18.71 Gb and a unitig N50 length of 3.97 Mb, which represents the best contiguity. Subsequently, a method of ‘global clustering and then local multiple iterative clustering’ (Hu *et al*., 2022) with the assistance of Hi‐C data and the diploid reference genome (Gong *et al*., 2022; Lin *et al*., 2022) were applied to assign and scaffold the unitigs to different homologous chromosomes. For each set, three homologous chromosomes were generated by clustering and ordering the contigs, using Hi‐C data. These analyses resulted in a total of 8.81 Gb of sequences (91.17% of the assembly), which were clustered into 45 chromosomes with a super‐scaffold N50 length of 180.00 Mb. Finally, after further removing plasmid and redundant sequences, gap filling, telomere patching and repeat‐aware polishing with HiFi and Illumina reads, the ultimate Changlin40 assembly comprised 1899 scaffolds (N50 = 180.00 Mb); of which the 45 pseudochromosomes comprising 15 homologous groups with three members each to representing all chromosomes occupied 91.22% of all 8.80 Gb assembly genome sequences (Table 1, Figure 1c; Figure S4; Table S4). Moreover, the robust interaction signals observed between the homologous chromosomal groups of Changlin40 (Figure 1c; Figure S4), coupled with the high frequency of homozygous genome sequences (Figure S2) and cytological observations (Figure S3), suggest that it possibly represents an auto‐hexaploid species.

**Table 1:** Metrics of the oil‐Camellia genome assembly

Genomic features	Changlin40	*Camellia lanceoleosa* (Gong *et al*., 2022)	*Camellia oleifera* (Lin *et al*., 2022)
Ploidy	6	2	2
Assembly statistics			
Total assembly size of contigs (Mb)	8800	2999	2891
Number of contigs	4411	3739	7312
Maximum contig length (Mb)	23.5	19.9	15.3
N50 contig length (Mb)	4.6	1.2	1.0
Number of scaffolds	1,899	790	2,143
N50 scaffold length (Mb)	180.0	186.4	185.4
Sequence on chromosomes (%)	91.22	91.8	91.3
Assembly‐BUSCO			
Complete (%)	96.7	95.5	92.2
Complete and single copy (%)	12.6	86.9	84.5
Complete and duplicated (%)	80.9	8.6	7.7
Fragmented (%)	1.6	1.7	2.4
Missing (%)	4.9	2.8	5.4
Annotated repetitive sequences (%)	78.48	69	80.63
Annotated protein‐coding genes	135 868	54 172	42 462
Annotation‐BUSCO			
Complete (%)	95.4	94.3	51.9
Complete and single copy (%)	29.4	79.4	39.8
Complete and duplicated (%)	66.0	14.9	12.1
Fragmented (%)	1.5	3.0	5.5
Missing (%)	3.1	2.7	42.6
LTR assembly index (LAI)	10.88	12.45	10.48

### Assessment and annotation of hexaploid Changlin40 genome

The completeness and accuracy of the Changlin40 were assessed using several independent analyses. First, according to data calculations, the size of the homologous chromosomal groups in Changlin40 is nearly three times that of the published diploid *Camellia lanceoleosa* (Gong *et al*., 2022) and *Camellia oleifera* (Lin *et al*., 2022) genomes (Table 1). Then, Illumina short reads and long HiFi reads were mapped to the assembled genome, and 92.68% and 99.91% of mapping rates were calculated indicating the high completeness of their sequence assembly. Genomic integrity was also evaluated using 1614 Benchmarking Universal Single Copy Orthologs (BUSCO) genes and found around 93.5% of them could be completely identified in the Changlin40 genome (Table 1), implying high completeness of the genome assembly. In addition, the annotation of long terminal repeats (LTRs) revealed an LTR Assembly Index (LAI) (Ou *et al*., 2018) score of 10.88, comparable to the scores in previously published genomes of diploid *Camellia oleifera* (Lin *et al*., 2022) and *Camellia lanceoleosa* (Gong *et al*., 2022) (Table 1). Interestingly, we also identified some telomere sequences in fragmentary scaffolds that were not anchored to chromosomes (Table S5), which may be unassembled chromosome ends. Likewise, the chromosomal interaction maps generated through Hi‐C data confirmed the correct order and orientation of all chromosomes. In addition, the Changlin40 assembly was in high synteny to the diploid *Camellia oleifera* and *Camellia lanceoleosa* reference genome sequences (Figure S5) also suggesting that the structure of the Changlin40 chromosomes was assembled correctly. Moreover, the base‐level accuracy and completeness for Changlin40 genome were further estimated by comparing k‐mers in the assemblies and HiFi reads with Merqury. The results showed that the consensus quality (QV) score and completeness of the assembly were 29.53 and 99.94% respectively. Taken together, these results show that this chromosome‐scale assembly of the Changlin40 genome exhibit higher reliability and quality.

**Figure 2:**
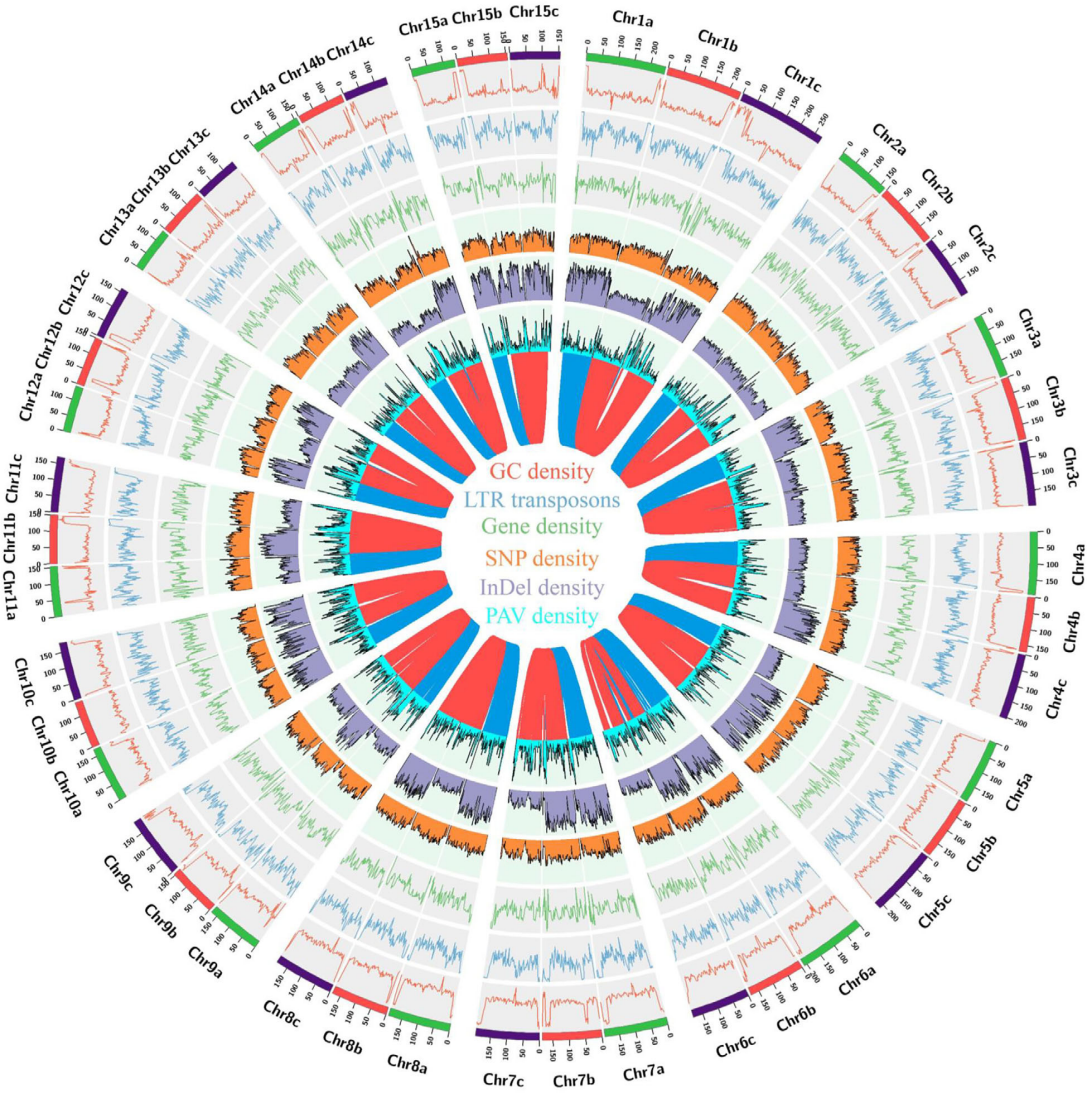
Overview of the assembled Changlin40 genome. Circular tracks from outside to inside indicate (a) the pseudomolecules; (b) GC content; (c) repetitive density; (d) gene density; (e) SNPs in each homologous chromosome group; (f) InDels in each homologous chromosome group; (g) distribution of PAV sequences in each homologous chromosome group. (h) The links in the centre show syntenic region found in each homologous chromosome group.

Subsequently, the genome repetitive sequences of the Changlin40 were identified using both homology‐based prediction and *de novo* identification. In total, approximately 78.48% (6.90 Gb) of the Changlin40 genome assembly was annotated as repetitive sequences, similar to diploid *Camellia oleifera* (Lin *et al*., 2022) (69%) and *Camellia lanceoleosa* (Gong *et al*., 2022) (80.63%) (Table 1 and Table S6). Among them, the LTR retrotransposons, mainly *Gypsy type* (33.53%) and *Copia type* (5.87%), are predominant in the Changlin40 genome (Table S6). Then, based on large‐scale RNA‐seq data including short reads of 24 biological samples spanning across different tissues and developmental stages of floral organs, and long reads of the isoform sequencing (Iso‐seq; Table S7), a comprehensive annotation was performed for the Changlin40 genome applying repeat‐masked genome combining *ab initio* prediction, homology‐based prediction and RNA‐sequencing‐assisted prediction. After integrating results of different software output by EvidenceModeler (Haas *et al*., 2008) and filtered out low‐quality gene models, a total of 135 868 protein‐coding genes were predicted, with an average length of 3936 bp (Table 1; Table S8). Among them, approximately 89.75% of the genes are located on 45 chromosomes, with an average of 2709 (SD = 653) genes per chromosome, which are 2.5 and 3.2 times the number of annotated genes in published diploid *Camellia lanceoleosa* and *Camellia oleifera* genomes (Table 1). Further evaluation, using BUSCO, indicated that 95.2% of plant‐conserved orthologues were fully identified in the Changlin40 genome assembly (Table 1), which is highly comparable with the annotations of the *Camellia lanceoleosa* and *Camellia oleifera* assemblies. Of the functionally annotated genes, 93.03% were predicted based on information from the NR (non‐redundant, NCBI), Swissprot, InterPro, Pfam and KEGG databases. Furthermore, the distribution of genes and repeats along the chromosome shows an opposite trend, with high gene and low repeat densities at the distal regions of the chromosome (Figure 2).

### Genomic variation and homologous chromosome diversity of oil‐Camellia

**Figure 3:**
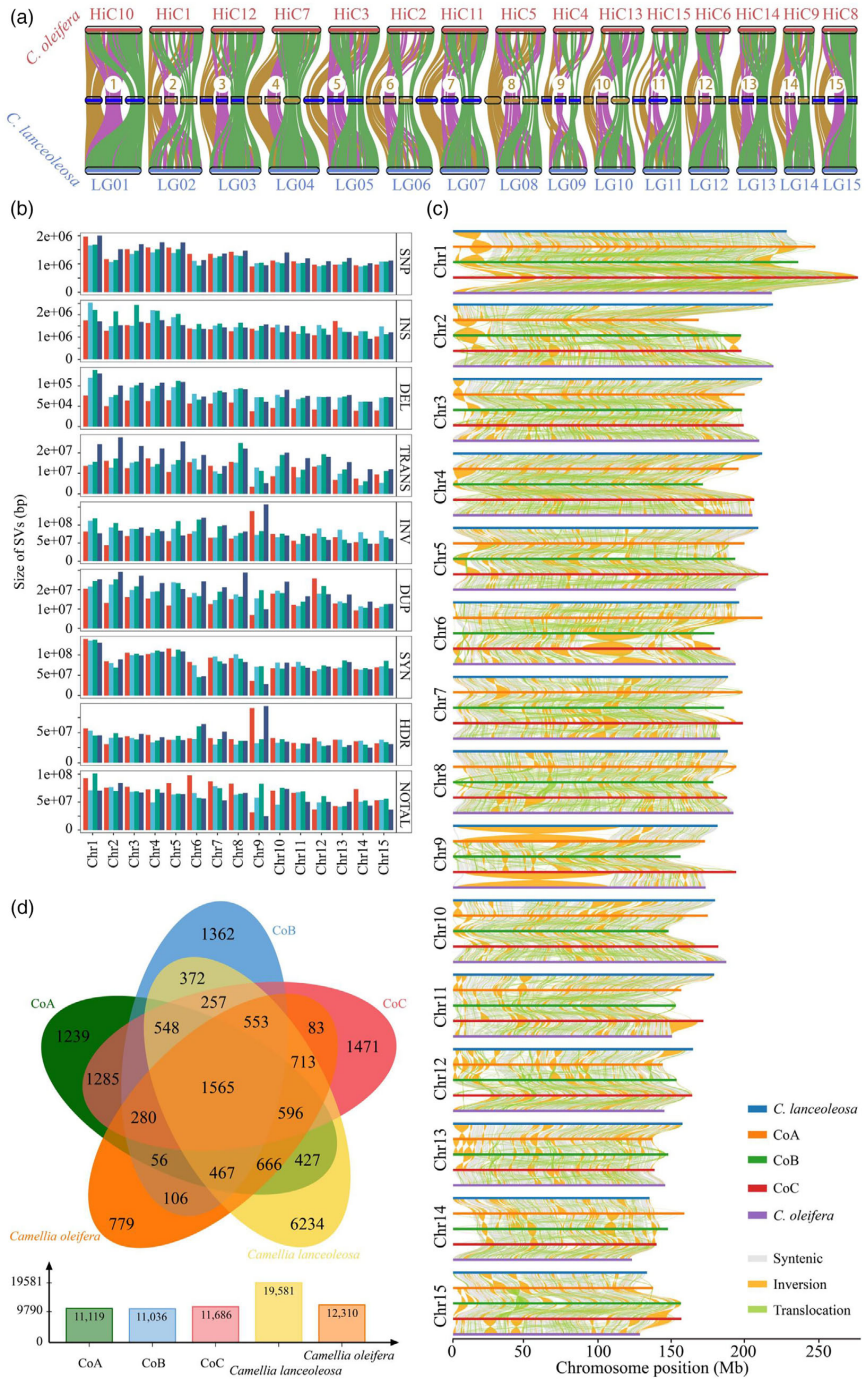
In‐depth analysis of the hexaploid Changlin40. (a) Genome‐wide syntenic relationships among two diploids *Camellia oleifera* and *Camellia lanceoleosa*, as well as the hexaploid Changlin40. (b) The distribution of structural variation size between the hexaploid Changlin40 and diploid. (c) Structural rearrangements between each homologous chromosome group. (d) Venn diagram of gene families of hexaploid Changlin40 genome and the other two diploid genomes.

To investigate the genomic divergence, the genomic synteny was assessed with homologous chromosome group of hexaploid and diploid (*Camellia oleifera* and *Camellia lanceoleosa*) at whole‐genome orthologous gene levels, and results show that two diploid genomes are highly homologous with those three homologous chromosome group of hexaploid with high genes coverage (Figure 3a). When further exploring the diversity between homologous chromosome groups with diploid, a total of approximately 20 million single nucleotide polymorphisms (SNPs) and 2 million insertions/deletions (InDels) were found to be distributed across different chromosomes (Figure 3b,c; Figure S6). Additionally, there were 51 166 translocations and 145 576 inversions identified, spanning a cumulative length of 277.9 Mb and 1.2 Gb, respectively, representing 9.6% and 42.5% of the diploid genome size (Figure 3b,c; Figure S6). Furthermore, approximately 312.3 Mb of duplicated regions and 650.1 Mb of highly divergent regions were observed, alongside 1.3 Gb and 1.0 Gb of syntenic and non‐aligned regions, respectively (Figure 3b,c; Figure S6). However, these variations were relatively low within the hexaploid homologous chromosome group (Figure 3b,c; Figure S6). This result suggests that all homologous chromosome groups exhibit a high degree of similarity, consistent with the genomic characteristics of their hexaploid nature. In addition, the extremely high similarity between all homologous chromosome groups also suggested that they were recently inherited from a common ancestor, which will result in regions that are identical by descent (IBD) between these homologous chromosome groups (Figure 3c). Interestingly, we also found that compared to *C. lanceoleosa*, *C. oleifera* CON has a higher number and proportion of structural variations mentioned above (Figure 3b,c; Figure S6). These observations suggest that Changlin40 may have a relatively distant genetic relationship with *C. lanceoleosa* and a closer genetic relationship with *C. oleifera*. Overall, the distribution of genomic variation across the chromosomes was uniform, without obvious mutation hotspots (Figure 3c and Figure 2). When further clustering of homologous genes between hexaploids and diploids, it was found that approximately 1565 gene families were shared by diploid and hexaploid homologous chromosomal groups, while 4560 gene families were shared by hexaploid and two diploids (Figure 3d).

### Comparative genomics and whole‐genome duplication in hexaploid oil‐Camellia

**Figure 4:**
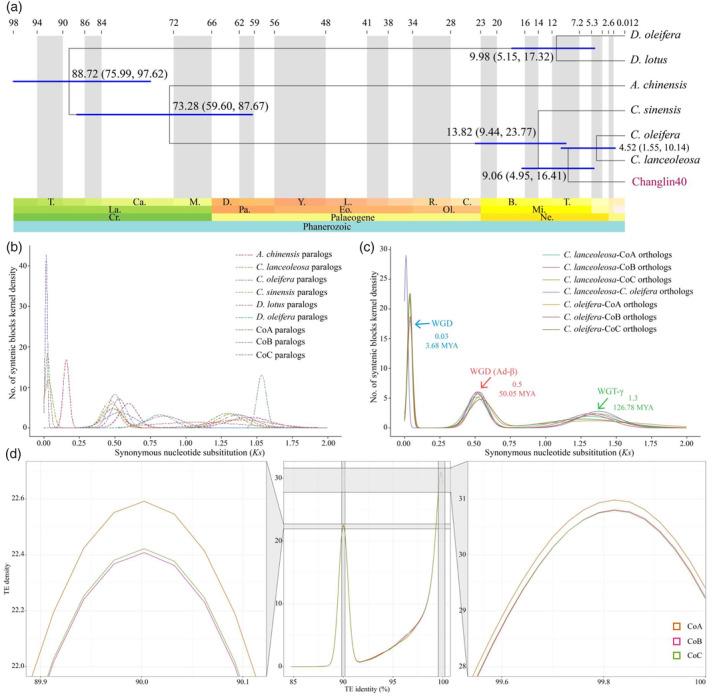
Genome evolution of the hexaploid Changlin40. (a) Phylogenetic tree showing the evolutionary relationship of oil‐Camellia and other species. Divergence timings and the supported bootstrap values were labelled on the tree. (b, c) Synonymous substitution rate (*Ks*) density distributions of syntenic paralogues (b) and orthologues (c), with coloured lines representing comparisons among species. (d) The distribution of sequence divergence rates among transposable elements (TEs) present in the homologous chromosome of Changlin40. The left and right panels display a partially magnified view of the two peaks in TE distribution.

To further clarify the evolutionary position of oil‐Camellia, we compared the protein‐coding genes of each homologous chromosome in Changlin40 with six other representative plant species, namely two diploid Oleifera (*Camellia oleifera* and *Camellia lanceoleosa*), two diploid ‘*Kaki*’ (*Diospyros lotus* (Akagi *et al*., 2020) and *Diospyros oleifera* (Zhu *et al*., 2019)), one other diploid *Camellia sinensis* (Zhang *et al*., 2021) and *Actinidia chinensis* (Pilkington *et al*., 2018), to identify orthologous groups. On the basis of the identified single‐copy orthologous genes, we inferred that the divergence between *C. sinensis* and oil‐Camellia took place after the speciation of *A. chinensis*, with the approximate times for the two events being 13.82 and 73.28 MYA, respectively (Figure 4a). The diversification of hexaploid Changlin40 with diploid *C. lanceoleosa* and *C. oleifera* occurred at approximately 9.06 MYA (Figure 4a). Subsequently, the evolutionary dynamics of gene families were analysed and functional exploration of Changlin40‐specific gene families indicated that KEGG pathways such as phenylalanine metabolism, fatty acid elongation, plant–pathogen interaction, flavonoid biosynthesis, alpha‐linolenic acid metabolism and carbon metabolism were significantly enriched (corrected *P*‐value < 0.05) in the Changlin40 genome (Figure S7). Interestingly, we found that gene families that participate in ABC transporters pathway were contracted in Changlin40 genome. While, the encoding enzymes associated with the biosynthesis of these key metabolites, such as meristem maintenance, regulation of root meristem growth, ubiquitin‐like protein ligase binding and amino acids metabolism, were also expanded, often in a species‐specific manner, in the Changlin40 genome (corrected *P*‐value < 0.05) (Figure S8).

It is well documented that WGD events have occurred frequently in the evolutionary history of flowering plants and generally shaped the evolutionary trajectory of genomes (Ren *et al*., 2018). The chromosome‐scale pairwise syntenic relationships within the Changlin40 genome (Figure 1c; Figure 2) and the genomic synteny between Changlin40 and diploid *C. lanceoleosa* and *C. oleifera* (Figure S5) supported the hexaploid origin of Changlin40. Indeed, the distribution of synonymous substitution (*Ks*) values of syntenic gene pairs within the homologous chromosome group of Changlin40 revealed an ancient whole‐genome triplication (WGT‐γ) event (*Ks* = ~ 1.3 and 126.78 MYA) that shared by the core eudicots along with a WGD (Ad‐β) event (*Ks* = ~0.5 and 50.05 MYA) that specific to the Theaceae family (Figure 4b). Additionally, it is noteworthy that all homologous chromosome groups possess a similar *Ks* peak value (Figure 4b,c). Likewise, the distribution of *Ks* values observed in diploid *C. lanceoleosa* and *C. oleifera* also displayed two peaks (Figure 4b,c) that are consistent with both Changlin40 and previous report (Gong *et al*., 2022; Lin *et al*., 2022). Moreover, we also identified a Camellia genus‐specific recent WGD event at *Ks* peak 0.03 (3.68 MYA; Figure 4b,c).

Furthermore, transposable elements (TE) were collected from homologous chromosomes and assessed their divergence rates (Figure 4d). The result showed that TE sequence divergence in homologous chromosome displays high degree of similarity, suggesting the consistency of TE evolutionary rate between them. Intriguingly, we also identified TE contents in homologous chromosome with identity approaching 90% and 100%, which formed a ‘bubble’ peak in the TE divergence profile (Figure 4d), which suggested that TE substitution rates in homologous chromosome differentiated. Moreover, the two peaks of TE identity observed can potentially reflect the two WGD events detected in the *Ks* distribution of the paralogue gene pairs as described previously (Figure 4b,c). Among them, TE arising from ancient WGD events have undergone substantial variation during evolution, while TEs replicated from recent WGD events still maintain high sequence similarity.

### Systematic multi‐omics integration (MOI) approach for discovery genes involved in seed oil biosynthesis

**Figure 5:**
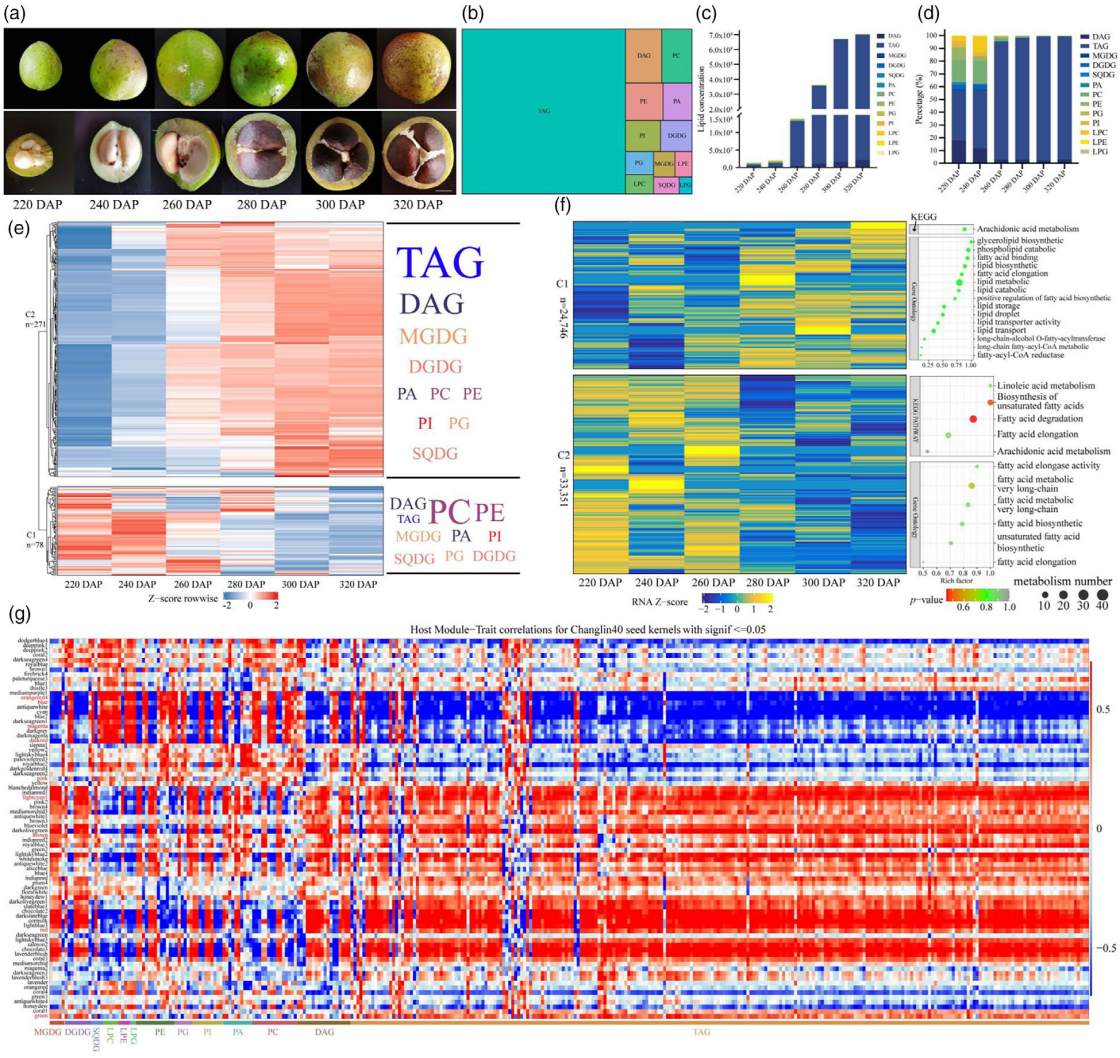
Elucidation of the biosynthetic pathway for Camellia oil in Changlin40. (a) Six developmental stages of seed kernels in Changlin40. DAP, days after pollination. Bar, 1 cm. (b) Treemap indicating the classification and relative content of oil‐related metabolites in seed kernels of Changlin40. (c, d) The total (c) and relative (d) content of oil‐related metabolites in seed kernels of Changlin40 at six different developmental stages. (e) Overview of oil‐related metabolome throughout seed kernels developmental stages of Changlin40. Metabolites in Clade I were primarily accumulated during the initial stages of seed kernel development; metabolites in Clade II were primarily accumulated during the later stages of seed kernel development. (f) Transcriptomic data are divided into two clades during Changlin40 seed kernel developmental stages. Genes in group I are highly expressed during the initial stages of seed kernel development; and genes in group II are highly expressed during the later stages of seed kernel development. (g) Heatmap showing module–oil correlations. Each column corresponds to a module indicated by different colours. Each row corresponds to an oil compound. Red colour indicates a positive correlation between the cluster and the seed kernels developmental stages. Blue colour indicates a negative correlation.

Camellia oil is a naturally high‐grade edible vegetable oil extracted from the kernels of mature seeds of *C. oleifera*, and its by‐product tea withered cake is also widely used in chemical industry, medicine, pesticide, feed, biological protein and other industrial fields (Wu *et al*., 2022a). To investigate the genetic regulation mechanism underlying the biosynthesis and metabolism of Camellia oil, we performed a comprehensive investigation through metabonomic and transcriptomic analyses of seed kernel samples at six developmental stages (220, 240, 260, 280, 300 and 320 DAP, day after pollination), obtained from a 15‐year‐old Changlin40 tree using liquid chromatography–tandem mass spectrometry (LC–MS/MS) and next‐generation sequencing technology (Figure 5a). A total of 349 glyceride metabolites were identified, which can be categorized into 13 distinct lipid types of molecular species with different carbon chain lengths and saturations (Figure 5b; Table S9; Note S1.1). The average total lipid content of the seed kernels demonstrated a distinct pattern with an initial period of rapid increase followed by a slower increase (Table S10). Specifically, there was a slight increase observed during periods S1 to S2 and S5 to S6, but these changes were not statistically significant (*P* > 0.05). However, during the period from S2 to S5, there was a highly significant increase (*P* < 0.05) in the total lipid content (Figure 5c; Table S11).

Triacylglycerols (TAGs) are the most abundant lipid species in Changlin40 seed kernels, constituting over half of the total lipids at different developmental stages of seed kernels. Galactolipids (MGDG and DGDG) and SQDG account for a relatively lower proportion of total lipids during seed kernel development, with their mass fraction progressively decreasing. Phospholipids (PA, PC, PE, PG and PI) and three lysolipids (LPC, LPE and LPG) accounted for approximately 38.08% of the total lipids at the early stage of seed kernel development (220 DAP and 240 DAP) but rapidly declined to 0.69% at the mature stage of seed kernel development (320 DAP) (Figure 5d).

Furthermore, based on their accumulation at different developmental stages, these metabolites were further divided into two groups (Figure 5e). The glycerides in group I included 6 DAG, 6 PA, 16 PC and 11 PE and a few molecules of TAG, PI, PG, MGDG, DGDG and SQDG (Figure 5e). Among these glycerides, the TAG reservoir primarily consists of C14:0 (myristic acid), C16:0 (palmitic acid), C16:1 (palmitoleic acid) as well as other medium‐ and long‐chain saturated and monounsaturated fatty acids. This phenomenon can be attributed to the preferential accumulation of 16:0 in the early stages of DAG production, as DAG serves as a precursor for TAG biosynthesis. PA serves as a precursor for the biosynthesis of various lipids and a second messenger for lipid signalling. During the early stages, PA predominantly accumulates C36 molecules, which provide an ample supply of precursors for the biosynthesis of PE and PC molecules. Notably, C36 molecules also represent the primary constituents within the PE and PC molecules. These lipid metabolites of group I exhibit prominent accumulation during the initial stages of seed kernel development and then gradually decrease. The glycerides within group II primarily accumulated during the later stages of seed kernel development and were consistent with the accumulation trend of total lipids in seed kernels of Changlin40 (Figure 5c). This group includes a majority of TAG and DAG molecules, six molecules of MGDG, four molecules of DGDG, as well as a small number of PA, PC, PE, PI, PG and SQDG molecules (Figure 5e). TAG serves as the primary form of energy storage in plants and is predominantly stored in seeds and fruits. During the later stages of Changlin40 development, a substantial number of TAG molecules accumulate, with notable compositions including C18:0 (stearic acid), C18:1 (oleic acid), C18:2 (linoleic acid) and C18:3 (linolenic acid). These fatty acids exhibit preferential accumulation during fruit ripening and are likely to become the major types of fatty acids that influence the quality of Changlin40 fruits. MGDG and DGDG are the principal lipids found in plastids, collectively known as galactolipids, and are closely associated with photosynthetic characteristics. Within MGDG and DGDG, C36 molecules also dominate. In addition, the biosynthesis of MGDG and DGDG is dependent on PA as the precursor, and the abundant presence of C36 molecules synthesized may be a significant factor contributing to the early‐stage accumulation of C36 molecules by PA. The results indicate discernible disparities in the customary lipid metabolites of Changlin40 during its early and later developmental stages. The main composition of lipid metabolites undergoes a shift, transitioning from phospholipids (PA, PC and PE) at the early stages to triglycerides (TAG) in the later stages. Noteworthy is the ubiquitous presence of C18 fatty acid chains in both TAG and principal phospholipids. The periodic accumulation of these substances provides novel insights into the key lipids that impact the quality of Changlin40.

Furthermore, the transcription pattern during seed kernel developmental stages was also investigated and these genes could be divided into two groups (Figure 5f) as found for the accumulation pattern of oil‐associated metabolites. Group I genes (43%) were specifically expressed during the initial stages of seed kernel development. Notably, these genes demonstrated significant enrichment and high expression levels in pathways related to the biosynthetic of unsaturated fatty acids, fatty acid degradation and elongation processes (Figure 5f). Group II genes (57%) were mainly expressed during the later stages of seed kernel development and the corresponding glycerolipid biosynthetic pathway genes showed significant enrichment and high expression. (Figure 5f). Overall, both the metabolome and transcriptome data showed significant developmental specificity at different stages of seed kernel development in Changlin40.

To gain further insight into the regulation of the metabolic changes throughout Changlin40 seed kernel development, weighted gene co‐expression network analysis (WGCNA) was performed to investigate the co‐expression networks. A total of 80 co‐expression modules were identified based on their similar expression patterns (Figure S9). The heatmap of module–trait correlations indicated that the accumulation of transcripts for the total of nine modules, including blue, brown, dark red, green, lightcyan1, magenta, orangered4, pink and red, was correlated with oil‐associated metabolites including PA, PC, DAG and TAG, which preferentially accumulated during the later stages of seed kernel development (Figure 5g). These results indicate that genes in these nine modules are mainly associated with Camellia oil changes during seed kernel development of Changlin40.

### Generation of camellia oil molecular regulatory networks in Changlin40

**Figure 6:**
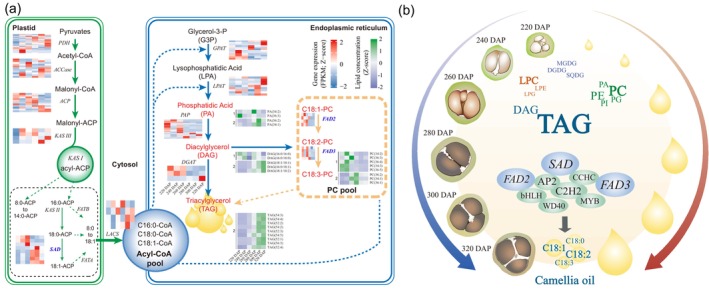
The regulatory network of key lipid metabolites in Changlin40. (a) The metabolic pathway for lipid. The red–blue heatmap represents the expression levels of corresponding catalytic genes at different seed kernel developmental stages, while the green–purple heatmap represents the content of corresponding metabolites at different seed kernel developmental stages. PDH, Pyruvate dehydrogenase; ACCase, Acetyl‐CoA carboxylase; ACP, Acyl carrier protein; KASIII, Beta‐ketoacyl‐(acyl‐carrier‐protein) synthase III; FATB, Fatty acyl‐ACP thioesterase B; KASII, Beta‐ketoacyl‐(acyl‐carrier‐protein) synthase II; SAD, Stearoyl‐ACP desaturase; FATA, Fatty acyl‐ACP thioesterase A; LACS, Long‐chain Acyl‐CoA synthetase; GPAT, Glycerol‐3‐phosphate acyltransferase; LPAT, Lysophosphatidic acid acyltransferase; PAP, Purple acid phosphatase; DGAT, Diacylglycerol acyltransferase; FAD2, Fatty acid desaturase 2; FAD3, Fatty acid desaturase 3. (b) Schematic of Camellia oil biosynthesis in Changlin40 seed kernel.

In Changlin40 seed kernels at different developmental stages, lipids, primarily TAG and DAG, constitute the main components. Among them, TAG represents the majority (Figure 5b). During the development of Changlin40 seeds, Camellia oil biosynthesis mainly involves the utilization of glycerol 3‐phosphate (G3P) and acyl‐CoA as precursors. The enzymatic action of G3P acyltransferase (GAPT) leads to the formation of lysophosphatidic acid (LPA), which further undergoes acylation by lysophosphatidic acid acyltransferase (LPAT) to form phosphatidic acid (PA). PA is then converted to DAG through the action of phosphatidate phosphatase (PAP), and ultimately, DAG is converted to TAG with the aid of DAG acyltransferase (DGAT) (Figure 6a). Moreover, the total carbon number (CN value) of the acyl groups in TAG of Changlin40 seeds is predominantly C54, with the highest content observed in TAG species such as TAG54:3, TAG54:4 and TAG54:2. Following this, C52‐containing TAG species (TAG52:2 and TAG52:3) constitute a significant portion. TAGs with CN values of C50 and C56 also contribute to the overall lipid composition (Table S9).

From overview of the component content of Camellia oil at the developmental stages of Changlin40 seed kernels, it is evident that the exceptionally abundant presence of unsaturated fatty acids represents the primary determinant of the high‐quality seed oil in Changlin40 (Figure 5c). The lipid composition of Changlin40 predominantly consists of unsaturated fatty acids, including C18:1 and C18:2, followed by saturated fatty acid C16:0 and unsaturated fatty acid C18:3 (Table S9). The content of unsaturated fatty acids in Camellia oil exceeds 90%, with the highest proportion of monounsaturated fatty acid C18:1 (Figure S10). The pathway analysis shows that, in the plastids, acetyl‐CoA serves as the precursor for the biosynthesis of saturated fatty acids such as palmitic acid (C16:0) and stearic acid (C18:0) through the enzymatic activity of fatty acid synthase. C18:0 then undergoes catalysis by *stearoyl‐acyl carrier protein desaturases* (*SADs*) to form oleic acid (C18:1). Further desaturation of C18:1 is facilitated by *fatty acid desaturase 2* (*FAD2*), resulting in the formation of linoleic acid (C18:2). Subsequently, C18:2 can be further desaturated by *FAD3* to yield linolenic acid (C18:3). The availability of the high‐quality Changlin40 genome assembly presents us with an invaluable opportunity to delve into the genetic mechanisms that underlie its exceptional qualities. Notably, the *SAD* exhibits significant high expression throughout all developmental stages of the seed kernel in Changlin40, with a discernible shift in the dominant gene set during the initial and later stages of seed kernel development (Figure 6a). Conversely, *FAD2* and *FAD3* demonstrate predominantly high expression during the initial stage (Figure 6a), aligning with the period of rapid accumulation of C18:2 and C18:3 unsaturated fatty acids (Figure S10).

To generate the regulatory network associated with Camellia oil metabolism, we examined the structural genes involved in Camellia oil metabolic pathway identified in the aforementioned nine modules (Figure 5g). In the seed kernels at different developmental stages, we have identified genes involved in TAG metabolism. These genes comprise *LACS*, *GPAT*, *LPAT*, *PAP*, *DGAT*, *FAD2* and *FAD3* genes (Figure 6a). Furthermore, these genes exhibit biased expression patterns across developmental stages of the seed kernels (Figure 6a). This indicates that the expression levels of these genes vary considerably as the kernels undergo development. Such biased expression reflects the importance of these genes in regulating TAG metabolism at different stages of kernel development. In addition, we selected two key genes involved in the biosynthesis pathway of Camellia oil, *SAD* and *FAD3*, for comparative genomic analysis. Undoubtedly, compared to two diploid species, these two genes have the highest copy number in Changlin40. Subsequently, evidence of selection across *SAD* and *FAD3* genes was tested using a multispecies alignment in HyPhy with the datamonkey webserver (https://www.datamonkey.org/) (Delport *et al*., 2010). In the sequence‐level analysis of *SAD* expansion, most conserved regions (108 sites) were under strong purifying selection, with seven sites showing episodic diversifying selection (Figure S11a). Phylogenetic analysis showed that the *SAD* gene demonstrates a distinct species‐specific clustering pattern in both diploid and hexaploid Changlin40 (Figure S11b). This observation suggests potential functional divergence within the gene. For the *FAD3* gene, the same analysis indicates that 28 conserved sites were under strong purifying selection, with 24 sites showing episodic diversifying selection (Figure S11c). Phylogenetic analysis showed that the *FAD3* gene is grouped into three distinct clusters, with each cluster encompassing gene sequences from both diploid and hexaploid Changlin40. This indicates that the *FAD3* gene is either functionally conserved or exhibits a certain co‐evolutionary trend in the biosynthesis of Camellia oil across the diploid and hexaploid Changlin40 (Figure S11d).

By correlating the patterns of transcript accumulation and the potential binding affinity for the promoters of metabolic structural genes associated with lipid biosynthesis (Figure 6a), we have identified 45 structural genes and 240 TFs (Figure S12) including *AP2*/*EREBP*, *C2H2*, *WD40*, *CCHC(Zn)*, *MYB* and *bHLH* whose expression was highly correlated with the TAG‐metabolizing structural genes in the aforementioned nine modules (Figure 5g) and formed a correlation network (Figure S13). Notable, this correlation network demonstrates the interplay and regulatory relationships between the TFs and the structural genes associated with lipid metabolism. Moreover, it underscores the inherent capacity of these TFs to modulate gene expression pertaining to TAG metabolism.

In summary, our comprehensive analysis of the genome, transcriptome and metabolome has revealed the dynamic regulation of Camellia oil biosynthesis by different key genes (Figure 6b). During the developmental stages from 220 DAP to 320 DAP, the morphology of Changlin40 seeds gradually developed from early stage to complete maturity and showed significant internal structural changes. Concurrently, this developmental process coincides with the accumulation of different oil components, particularly after 300 DAP, where Camellia oil content experiences a notable surge (Figure 5c). During this phase, key genes (*SAD*, *FAD2* and *FAD3*) and transcription factors (*AP2*, *C2H2*, *CCHC*, *MYB*, *bHLH* and *WD40*), which play crucial roles in governing lipid biosynthesis and accumulation. Overall, the developmental changes, lipid biosynthesis pathways and gene regulatory networks provide a scientific foundation for furthering quality enhancements and applications of Changlin40.

## Discussion

Here, we first ever reported a chromosome‐scale reference genome assembly of the hexaploid species, oil‐Camellia, one of the most important woody edible and industrial oil tree species. Currently, although three diploid *Camellia oleifera* (Gong *et al*., 2022; Lin *et al*., 2022; Shen *et al*., 2022) genomes have been sequenced and assembled, considering the rich species diversity of *Camellia oleifera* and the scarce systematic research foundation (Kondo, 1977; Ye *et al*., 2023), there is no information regarding the origin and the direct diploid progenitors of hexaploid oil‐Camellia. Therefore, future studies are needed to clearly elucidate the hexaploid origin of Changlin40. Nonetheless, the high‐quality genome assembly of hexaploid Changlin40 provides a crucial resource for future functional and comparative genomic studies and for facilitating the development of Camellia oil.

To date, only a limited number of allohexaploid plant genomes have been sequenced (Peng *et al*., 2022; Song *et al*., 2023), and only a few auto‐tetraploid plants have undergone genome assembly (Bao *et al*., 2022; Chen *et al*., 2020; Liu *et al*., 2024; Sun *et al*., 2022). Here, the chromosome‐scale hexaploid genome assembly allows us to identify the genomic sequence differentiation. Indeed, despite oil‐Camellia Changlin40 exhibits auto‐polyploid genomic characteristics, our comparative genomic analysis still revealed a high proportion of sequence differences and structural rearrangements between the homologous chromosomes (Figures 2 and 3), which were highly probable introduced via crossing‐related genotypes during breeding or introduced at the initial stage of polyploidy speciation and retained in the subsequent evolutionary process (Sun *et al*., 2022). In addition, polyploidy has been widely recognized as a major force in plant evolution and speciation. It can occur through several mechanisms, including WGD events and hybridization between species and somatic doubling (Mason and Wendel, 2020). Indeed, the comparative analyses reveal that hexaploid oil‐Camellia Changlin40 has undergone one ancient WGT‐γ event that is shared by all core eudicots and one recent WGD (Ad‐β) event that is shared by Camellia and Actinidia (Figure 5). These findings were consistent with previous studies in potential diploid ancestors *Camellia oleifera* (Lin *et al*., 2022) and *Camellia lanceoleosa* (Gong *et al*., 2022), as well as other *Camellia* species (Xia *et al*., 2017).

Oil‐Camellia species produce diverse types of natural oil products. The dataset of metabonomic and transcriptomic provides a useful resource for the clarification of the metabolic pathways of key oil compounds not yet deciphered in Changlin40. Combining these transcriptomic and metabolomic resources allows the identification of key structural and regulatory genes involved in oil‐related metabolism in Changlin40. Specifically, metabolomic analysis revealed a dynamic shift in the lipid metabolite composition of Changlin40, transitioning from phospholipids (PA, PC and PE) in the early stage to TAGs in the later stage. This transformation is believed to be influenced by the intricate interplay between PA, a pivotal lipid intermediate involved in early cell proliferation and membrane lipid biosynthesis, and the subsequent accumulation of substantial quantities of TAGs during the later stage (Hung *et al*., 2016). In addition, WGCNA identified nine modular genes intricately involved in the biosynthesis and metabolism of Camellia oil. Among these, 45 structural genes, including *DGAT* and *FAD2*, demonstrated high correlations with the expression of 240 associated TFs (Gong *et al*., 2022; Lin *et al*., 2022). Intriguingly, the interaction network analysis between these components offers novel insights for comprehensively understanding the intricate landscape of fatty acid metabolism in Camellia oil (Figure 6b).

In conclusion, the genomic resources we present here can help mine hub genes governing important traits in oil‐Camellia and contribute to the study of oil‐Camellia Changlin40 evolution. Additionally, the hexaploid oil‐Camellia genome sequence can serve as a vital resource for studying the genetic bases of these major plant metabolic pathways and for germplasm utilization to breed improved oil‐Camellia cultivars. The hexaploid genome of Changlin40 may help to guide assembly of other large complex polyploid genomes with high heterozygosity and uncertain origins.

## Methods

### Plant growth and genomic sequencing

The cultivar ‘Changlin40’ of hexaploid *Camellia oleifera* was obtained from the Hubei Academy of Forestry (Figure S1; 114°37′ E, 30°52′ N; Huanggang, Hubei, China) and used for the genome sequencing.

For Illumina and PacBio sequencing, young leaves were collected for high‐molecular‐weight DNA (hmwDNA) extraction and then prepared and sequenced on the Illumina HiSeq 2500, PacBio Sequel II platform respectively. For the Hi‐C experiment, a Hi‐C library was constructed by chromatin extraction and digestion, DNA ligation, purification and fragmentation using the standard protocol. DpnII was used to digest genomic DNA. Then, the Hi‐C libraries were sequenced with 150‐bp paired‐end reads using Illumina HiSeq.

For the expression atlas sequencing, diverse tissues, including stem, leaves, flower and zygotic embryo, with different development stages, representing the major organ systems, were collected and immediately frozen in liquid nitrogen, with three biological replications. The total RNA per sample was extracted and purified. After DNase treatment, RNA‐seq libraries were constructed and sequenced on the Illumina HiSeq 2500 platform with 150 bp paired‐end sequences according to the manufacturer's recommended protocol.

For the systematic multi‐omics integration analysis of seed oil biosynthetic, seeds were collected at 0, 4, 8 and 10 days after anthesis (DAA), representing different stages of seed oil accumulation in embryo. Subsequently, metabolomic and transcriptome analyses were performed on three biological replicates at each time point.

### Karyotype analysis

The root tips of cutting seedlings of Changlin40 were pretreated with 0.002 mol/L 8‐hydroxyquinoline for 5 ~ 6 h. After pretreatment, root tips were fixed with Carnoy solution for 12 h and treated in 0.075 mol/L KCl solution for 60 min. Subsequently, 1.75% cellulase and pectinase were enzymolysed for 120 min under 20 °C darkness and then cleaned with distilled water. After 30 min posterior hypotonicity with distilled water, and fixed with a new Carnoy solution for more than 30 min, smears, flame drying and carbo magenta staining were observed and photographed with a microscope (Olympus BX‐61, Japan). Choice of more than 30 chromosomes scattered good cell chromosome counting, scattered and choose good, clear chromosome karyotype analysis, split phase will use the proceeds of the chromosome image number and the measured value of the long arm and short arm, according to the data to carry on the homologous chromosome pairing combination, draw the karyotype model, from long to short order number. Finally, the average value of five cells was taken as the karyotype analysis parameter.

### Genome size estimation

Approximately 519.00 Gb Illumina reads were used to estimate the genome size. The k‐mer (*k* = 21) count was computed with Jellyfish (v.2.2.10) (Marçais and Kingsford, 2011) and was input to GenomeScope (v.2.0) (Ranallo‐Benavidez *et al*., 2020) with hexaploid mode.

### Genome assembly and quality assessment

#### Contigs assembly using HiFi and Hi‐C reads

The initial contigs assembly of the hexaploid genome was performed using hifiasm (v.0.16.1) (Cheng *et al*., 2021) with ‐f39 settings and paired‐end Hi‐C reads with the 379.58 Gb PacBio HiFi and 789.10 Gb Hi‐C reads, respectively, where the output consisting of unitigs (locally haplotype‐resolved contigs) was selected for further processing.

#### Chromosomal scaffolding

To construct a chromosome‐scale reference genome of hexaploid oil‐Camellia, the assembly method of ‘global clustering and then local multiple iterative clustering’ as used in Chinese pepper genome (Hu *et al*., 2022) was used to cluster and order the contigs into pseudo‐chromosomes. In brief, the trimmed Hi‐C reads were mapped to these contigs using BWA (v.0.7.17) (Li, 2013). Then, the uniquely mapped data were retained to perform assembly using LACHESIS (Burton *et al*., 2013) with parameters ‘CLUSTER_N = 90; CLUSTER_MIN_RE_SITES, 225; CLUSTER_MAX_LINK_DENSITY, 2; ORDER_MIN_N_RES_IN_TRUN, 105; ORDER_MIN_N_RES_IN_SHREDS, 105’. Based on the interaction signal strength within and between chromosomes, 90 groups, representing 90 pseudo‐chromosomes, were further subdivided into 15 subgroups with each containing six homologous chromosomes. Subsequently, a local multiple iterative clustering of six homologous chromosomes was performed using LACHESIS with parameters ‘CLUSTER_N = 6’. All 15 subgroups performed the above operations respectively. Finally, each three longest chromosomes were selected as representative of the homologous chromosome group within the Changlin40 genome. Subsequently, the assembled genome underwent manual scrutiny and refinement using Juicebox (v.1.11.08) (Durand *et al*., 2016).

To fill the gaps and polishing the genome, all HiFi reads were aligned to the genome using minimap2 (v.2.23) (Li, 2018) with ‘‐axe map‐hifi’ parameter. Then, non‐primary and chimeric read alignments were filtered using Samtools (v1.9) and ‘falconc bam‐filter‐clipped’ (https://github.com/PacificBiosciences/pbipa). A consensus assembly was produced through Racon (v.1.4.20; https://github.com/isovic/racon/). Finally, repeated four rounds of polishing of the genome were performed using Illumina reads and Pilon (v.1.23) (Walker *et al*., 2014) with parameters ‘‐‐mindepth 10 ‐‐changes ‐‐fix bases’.

#### Genome quality assessment

Multiple approaches were used to evaluate the quality of the assembled genomes. First, the conserved protein models from the lineage database embryophyta_odb10 were searched against genome by using the BUSCO (v.5.2.2) with parameters ‘‐‐augustus ‐‐long’ to evaluate the completeness of genome assemblies. Second, the short paired‐end reads and long HiFi reads were mapped to genome using Bowtie2 (v.2.3.5) (Langmead and Salzberg, 2012) and minimap2 (v.2.23) (Li, 2018) with default settings respectively. Subsequently, the genome coverage was counted and visualized. Third, the reference‐free base accuracy (QV) and completeness of each chromosome and the whole‐genome sequence of the assembly were evaluated using Merqury (v.1.3) (Rhie *et al*., 2020) with HiFi reads. Finally, the LAI (Ou *et al*., 2018) was used to evaluate the completeness in the more repetitive genomic regions.

### Genome annotation and assessment

#### Repeat prediction

As we described in the genome of Chinese pepper (Hu *et al*., 2022), a non‐redundant *de novo* repeat library of the genome was customized using RepeatModeler (v.2.0.1) (Flynn *et al*., 2020) and Extensive de novo TE Annotator (EDTA) (Ou *et al*., 2019). The potential protein‐coding genes were excluded by alignment to the Uniprot database using BLASTX (v.2.5.0), and unknown TEs were further classified using TEclass (v.2.1.3) (Abrusán *et al*., 2009). Then, the RepeatMasker (v.4.1.0) (Chen, 2004) was adapted to search and mask the genome against Repbase (Bao *et al*., 2015) and the species‐specific *de novo* repeat library. In addition, the repeat sequences with more than 10 monomers ‘AAACCT’ were identified as telomeres.

#### Gene annotation

Protein‐coding genes for Changlin40 genome were predicted using an evidence‐based annotation workflow by integrating evidence from transcriptomic data, homologue searches and *ab initio* prediction, following extensive and careful manual inspections and corrected. Transcriptomic data were generated by performing Illumina short RNA‐seq reads sequencing from stem, leaves, flower and zygotic embryo, and PacBio full‐length transcriptome sequencing from mixed of above organs. We used IsoSeq3 pipeline (https://github.com/PacificBiosciences/IsoSeq) with parameters ‘‐‐min‐rq 0.9’ to correct nonchimeric circular consensus sequences (CCSs) subreads. Lima (v.2.6.0) with the parameters ‘‐‐isoseq ‐‐peak‐guess’ was used to classify the full‐length reads. The refine and cluster models of isoseq3 were used to collect the final full‐length Iso‐seq transcripts. Subsequently, the full‐length transcripts from PacBio Iso‐seq reads were aligned to the genome by GMAP (v.2020‐03‐12) (Wu and Watanabe, 2005) with the parameters ‘‐‐min‐intronlength = 30 ‐‐trim‐end‐exons = 20’. The short RNA‐seq reads were aligned to genome by HISAT2 (v.2.2.1) (Kim *et al*., 2019) with the default parameters. The aligned reads from all organs were then merged, filtered and assembled by bamtools (v.2.5.1), portcullis (v. 1.2.4; https://github.com/EI‐CoreBioinformatics/portcullis) and StringTie2 (v.2.1.2) (Pertea *et al*., 2016) respectively. The TransDecoder (v5.5.0) was used to identify the coding sequence with default parameters. Additionally, protein sequences from *Arabidopsis thaliana*, *Camellia sinensis*, *Camellia oleifera* and *Camellia lanceoleosa* were used as protein evidence for homology‐based prediction with GeneWise (v.2.4.0) (Birney *et al*., 2004) using the default settings. The longest transcript was selected to represent each gene. ORFs, with premature stop codons, that were not multiples of three nucleotides long were also removed. Ab initio gene prediction was performed using GeneMark‐EP+ (v.4.0) (Besemer *et al*., 2001) and AUGUSTUS (v.3.3.1) (Stanke and Waack, 2003) with those selected proteins. After that, all gene predictions were integrated using the recommended settings of EVidenceModeler (EVM; v.1.1.1) (Haas *et al*., 2008) after removing TE‐related genes, pseudogenes and noncoding genes using TransposonPSI (v1.0.0; https://transposonpsi.sourceforge.net/) with the default settings.

#### Functional annotation of protein‐coding genes

The final annotation completeness of protein‐coding genes was evaluated against genome to lineage database embryophyta_odb10 BUSCO (v.5.2.2) using BUSCO (v.5.2.2) with parameters ‘‐‐augustus ‐‐augustus_species Arabidopsis ‐‐long’. Functional annotation of the predicted genes was performed by comparing their protein sequences against the GenBank non‐redundant (nr) and UniPort SwissProt databases. The domains and GO terms annotation of genes were performed with InterProScan (v.5.52) (Jones *et al*., 2014) with default parameters. The TFs and transcriptional regulators (TRs) from protein sequences were identified by mapping to 197 plant species database 18.12 (http://itak.feilab.net/cgi‐bin/itak/online_itak.cgi) through iTAK (v.1.7a) (Zheng *et al*., 2016), and then classified into different gene families.

The noncoding RNAs, including microRNAs, small nuclear RNAs and ribosomal RNAs, were annotated with the tool Infernal (v.1.1.2) (Nawrocki and Eddy, 2013) by searching the database Rfam (v.14.3) (Nawrocki *et al*., 2015). The ribosomal RNAs and transfer RNAs were identified using RNAmmer (v.1.2) (Lagesen *et al*., 2007) and tRNAscan‐SE (v.2.0) (Lowe and Chan, 2016) respectively.

### Synteny analysis among oil‐Camellia

Within each of the 15 homologous linkage groups, the chromosome‐level sequences of the three homologous were aligned to each other as well as to the recently assembled *Camellia lanceoleosa* and *Camellia oleifera* genome using minimap2 with parameters ‘‐axe asm20 ‐‐eqx’. For each pair, after filtering low‐confidence alignments, the alignments were provided to SyRI (v.1.6) (Goel *et al*., 2019), which searched for synteny, single‐nucleotide level differences as well as large‐scale structural variations (with ‐*k* ‐*F S*). Furthermore, we also used SnpEff (v.4.3) (Cingolani *et al*., 2012) to predict the functional effects of each SNP and InDel that may be under selection in the genome.

Syntenic gene pairs among oil‐Camellia, *Camellia lanceoleosa* and *Camellia oleifera* were also identified using JCVI (v.0.84) (Tang *et al*., 2008). The syntenic blocks for each pair species were identified using ‘jcvi.compara.catalog ortholog’ with a parameter of ‘‐‐cscore = 0.8’. The syntenic blocks were filtered using ‘jcvi.compara.synteny screen’ with parameters ‘‐‐minspan = 30 ‐‐simple’. Synteny pattern was detected using ‘jcvi.compara.synteny depth–histogram’.

### PAV analysis

To identify the PAV genes among homologous chromosomes, the predicted genes of each chromosome were prepared and input into SonicParanoid (v.1.3.5) (Cosentino and Iwasaki, 2018) to identify orthologous relationships among homologous chromosomes with default parameters. Those genes lacking homologue on any homologous chromosomes were defined as PAV genes. However, the PAVs in the genome sequences were identified through scanPAV (Giordano *et al*., 2018) with the default parameters and any PAVs that were shorter than 1000 bp were prudently filtered out as noise.

### Comparative and evolutionary analyses of the oil‐Camellia genome

#### Gene family analysis

Six species, included Changlin40, *Camellia lanceoleosa* (Gong *et al*., 2022), *Camellia oleifera* (Lin *et al*., 2022), *Camellia sinensis* (Xia *et al*., 2017) and *Actinidia chinensis* (Pilkington *et al*., 2018), for which high‐quality reference genomes were available and used for gene family clustering analyses. The longest transcript was selected to represent each gene. ORFs with premature stop codons that were not multiples of three nucleotides long, or encoded less than 50 amino acids, were also removed. Then, OrthoMCL (Li et al., 2003) was used to construct gene families based on all‐against‐all BLASTP alignment among the six species.

#### Phylogenomic analyses

To investigate the evolutionary position of oil‐Camellia, a phylogenetic tree was constructed using the 2237 conserved single‐copy genes among the six species. The conserved protein sequences of these single‐copy orthologues were aligned and extracted by using MAFFT (v.7.471) (Kazutaka and Standley, 2013) and Gblocks (v.0.91b) (Talavera and Castresana, 2007) and then concatenated to generate a supermatrix. The maximum‐likelihood phylogenetic tree was generated under the ‘PROTGAMMAAUTO’ model using RAxML (v.8.2.1264) (Stamatakis, 2014) to automatically determine the best reasonable tree by conducting 1000 bootstrap replicates.

#### Whole‐genome duplication and gene duplication analysis

To determine if there was a recent WGD in oil‐Camellia, we analysed the distribution of synonymous substitutions per site (*Ks*) for each paralogue in oil‐Camellia using WGDI (https://github.com/SunPengChuan/wgdi). The time of WGD event was estimated by the formula divergence date = *Ks*/(2 × *r*), where *r* refers to *Ks*/year rate of Camellia (Gong *et al*., 2022; Hu *et al*., 2022; Lin *et al*., 2022).

#### Estimation of divergence time

Divergence times were estimated using the MCMCTree with branch lengths estimated by BASEML in the PAML (v4.9) (Yang, 2007). The species tree constructed with the strictly single‐copy (SSC) orthologous genes from seven species was used as the input tree. The dates of the speciation events were obtained from study in *Camellia oleifera* (Lin *et al*., 2022) and *Camellia lanceoleosa* (Gong *et al*., 2022) genomes and subsequently used to calibrate the divergence time here. The Markov chain Monte‐Carlo analysis was repeated 10 000 000 times with 1000 steps. The divergence time was also corrected with the known calibration points sourced from Timetree (http://timetree.org/).

#### Gene family expansion and contraction analysis

The expansion and contraction of orthologous groups using computational analysis of gene family evolution (CAFÉ; v. 4.2) (De Bie *et al*., 2006) according to the difference in gene number of each orthologous group of each species. A family‐wise *P*‐value was set to 0.05 for each orthologous group based on a Monte‐Carlo resampling procedure to determine significance of expansion and contraction of orthologous groups in each gene family across species. The species‐specific Gene Ontology (GO) and Kyoto Encyclopedia of Genes and Genomes (KEGG) enrichment analyses were performed by KOBAS (http://kobas.cbi.pku.edu.cn/).

### Candidate gene for the oil biosynthesis in Changlin40

The homologues encoding oil biosynthesis in *Arabidopsis thaliana* (https://www.arabidopsis.org/) served as a reference to identify putative functional homologues in Changlin40 using BLASTP (v.2.2.28) with an e‐value of 1e‐10. For evolutionary analysis, we aligned protein sequences using MAFFT (v.7.471) (Kazutaka and Standley, 2013) with E‐INS‐I iterative refinement method and automatically trimmed by trimAl (v.1.1) (Silla‐Martínez *et al*., 2009). The fastTree (http://www.microbesonline.org/fasttree/) was then used to create maximum‐likelihood phylogenetic trees. The tree was visualized with iTOL (Letunic and Bork, 2021) (https://itol.embl.de/).

### Determination of seed oil content and composition using LC–MS/MS

Briefly, about 10 mg frozen dried samples were heated in 2 mL isopropanol with 0.01% butylated hydroxytoluene at 75 °C for 10 min to inactivate the lipases. Chloroform and methanol (2:1, v/v) were added for extraction. After several extractions, the combined extracts were washed with 1 M KCl to remove proteins and carbohydrates. The chloroform phase was taken out and dried under a nitrogen stream. Lipid extracts were dissolved in chloroform for lipidomic analysis. LC–MS/MS (multiple‐reaction monitoring mode) analyses were performed with a mass spectrometer QTRAP 4000 (ABSciex) mass spectrometer coupled to a liquid chromatography system (LC20A HPLC, Shimadzu) (Jouhet *et al*., 2017). Analyses were achieved in positive mode. Lipids were separated on an Accucore C30 (100 × 2.1 mm, particle size, 2.6 μm, Waters) using Eluent A and B solutions. Eluent A was formula water:methanol:acetonitrile:300 mM ammonium acetate = 20:20:20:1 (v/v/v/v), and eluent B was isopropanol:methanol:300 mM ammonium acetate = 180:20:3 (v/v/v). The gradient elution programme was as follows: 0–2 min, 25%–40% eluent B; 2–4 min, 40%–95% eluent B; and 4–18 min, eluent 95% B. The flow rate was 0.3 mL/min; 2 μL sample volumes were injected. The areas of LC peaks were determined using MultiQuant software (ABSciex) for relative quantification (Zhao *et al*., 2022).

### The construction of co‐expression networks

The trimmed paired reads of RNA‐seq through Trimmomatic (v.0.39) (Bolger *et al*., 2014) were mapped to the final Changlin40 genome using HISAT2 (v.2.2.1) (Kim *et al*., 2019) with default parameters. The expression abundance values (FPKM, TPM and expression count data) were calculated using StringTie (v.2.1.4) (Pertea *et al*., 2016) and also averaged the abundance values from the three biological replicates of each sample to obtain levels of gene expression.

For the construction of co‐expression networks, all RNA‐seq data from 14 samples described above (tissues of flowers at anthesis and pre‐anthesis, leaves and seedlings with different treatments) and required genes with transcripts per million (TPM) ≥ 1 in at least one of the samples to be included in the analysis. Then, a co‐expression network was generated using weighted gene co‐expression network analysis (WGCBNA) package in R. The co‐expression modules were obtained using default parameters, apart from the soft threshold power of 26, TOMtype was signed, mergeCutHeight was 0.25 and minModuleSize was 100. Subsequently, the Pearson correlation coefficients (PCCs) were calculated with the co‐expression modules and the fatty acid content was determined by LC–MS. Finally, the networks were visualized by Cytoscape (v.3.7.1, USA) (Shannon *et al*., 2003).

### Statistical analysis

All presented *P*‐values correspond to two‐sided *P*‐values. Correlation test was done using cor.test function in R (v.3.6.0). For RNA‐seq data analyses, differentially expressed genes were identified by the negative binomial test with *P*‐adj <0.05 and fold change >2.

## Funding

The study was supported by grants from the National Natural Science Foundation of China (31660427), Hubei Province Key R&D Plan (2021BBA260) and Central Guiding Local Science and Technology Development Special Project (2022BGE229) to Dr. Huaguo Zhu and National Science Fund of China for Distinguished Young Scholars (32325039) and grant from Hubei Hongshan Laboratory (2021hszd013) to Dr. Shuangxia Jin.

## Author contributions

S.J., X.H. and F.D. designed and supervised the research. Z.X. and F.W. performed the genome assemblies, annotation, transcriptome and phylogenetic analysis. H.Z., F.W., G.W. and L.H. collected materials for genome sequencing. X.G. performed karyotype analysis. W.C. and Q.L. used LC–MS/MS to determine the content and composition of seed oil. G.W., J.C., S.X, C.Y., F.X., F.L., L.W., X.C., X.T. and W.L. helped collect public data. Z.X., H.Z., G.W and L.H. wrote the manuscript with input from all other authors. K.L., X.Z., X.H., F.D. and S.J. edited the paper. All authors have read and approved the manuscript.

## Conflict of interest

No other conflict of interest is declared.

## Supporting information


**Data S1.** Supporting information.
**Note** Classification of glyceride metabolites from the seed kernel of Changlin40.
**Figure 1** The whole tree and fruit features of Changlin40.
**Figure 2** Estimation of Changlin40 genome size by 17‐mers depth distribution of 519 Gb raw Illumina sequence data. Two peaks are observed (49× and 104×): the first peak represents the unique part of the genome and the second peak represents the repetitive part of the genome.
**Figure 3** Meiosis process of the pollen mother cells in Changlin40.
**Figure 4** Hi‐C heatmap showing the chromosomal interactions of intra‐ and inter‐chromosomal within Changlin40 genome.
**Figure 5** Synteny of the Changlin40 genome comparison with *Camellia oleifera* (Lin et al., 2022) and *Camellia lanceoleosa* (Gong et al., 2022).
**Figure 6** The distribution of structural variation count between the hexaploid Changlin40 and diploid.
**Figure 7** KEGG pathways enrichment analysis of Changlin40‐specific genes. The color of circle represents the FDR (false discovery rate) in the hypergeometric test corrected using BH (Benjamini and Hochberg) method. The size of circle represents the gene count of the KEGG terms.
**Figure 8** KEGG pathways enrichment analysis of Changlin40 expansion genes. The color of circle represents the FDR (false discovery rate) in the hypergeometric test corrected using BH (Benjamini and Hochberg) method. The size of circle represents the gene count of the KEGG terms.
**Figure 9** Barplot showing co‐expression modules size identified by weighted correlation network analysis (WGCNA) across seed kernel development stages in Changlin40.
**Figure 10** Statistics pertaining to the contents of primary unsaturated and saturated fatty acids in Camellia oil during the developmental stages of Changlin40 seed kernels at relative (A) and absolute (B) levels.
**Figure 11** Analysis of gene involved in Camellia oil biosynthesis.
**Figure 12** The statistical distribution of structural gene and transcription factors contained in the lipid metabolism regulation network of seed kernel development in Changlin40.
**Figure 13** Network built based on the correlation between genes related to lipid metabolites. The structural genes associated with lipid metabolism were depicted as colored dots, while the TFs were represented by small cyan graphs. All modules significantly correlated with oil‐related metabolites were shown in this network.
**Table S1** Summary of Illumina reads (DNA) for Changlin40.
**Table S2** Summary of PacBio HiFi reads for Changlin40.
**Table S3** Summary of HiC reads for Changlin40.
**Table S4** Summary of Changlin40 genome assembly.
**Table S5** The telomere sequence in fragmentary scaffolds that were not anchored to chromosomes.
**Table S6** Summary of repeats in Changlin40 genome.
**Table S7** Summary of RNA‐seq and Iso‐seq reads of Changlin40.
**Table S8** General statistics of predicted protein‐coding genes in Changlin40.
**Table S9** The lipid concentrations of Changlin40 seed kernels in six different development stages.
**Table S10** Mean and standard deviation of total lipid concentrations of Changlin40 seed kernels in six different development stages.
**Table S11** The independent t‐test of samples from Changlin40 seed kernels in six different development stages.

## Data Availability

The assembly and annotation of *Camellia oleifera* var. Changlin40 were archived in the figshare (https://figshare.com/s/1a51c1909eab9cc0b603). The raw sequencing data used for *de novo* whole‐genome assembly are available from the NCBI under the BioProject accession number PRJNA993816. Transcriptome data of Illumina RNA‐seq and PacBio Iso‐Seq are available at the NCBI under the BioProject accession number PRJNA993817. Further details on data accessibility are outlined in the Supplementary Materials and Methods.
