# Supplementary material for: The complex hexaploid oil‐Camellia genome traces back its phylogenomic history and multi‐omics analysis of Camellia oil biosynthesis
Source: Plant Biotechnol J. 2024 Jun 24;22(10):2890–906. doi: 10.1111/pbi.14412 (PMC11536451; doi:10.1111/pbi.14412)
Supplement: Supplementary file 1 — Data S1. Supporting information. Note Classification of glyceride metabolites from the seed kernel of Changlin40. Figure 1 The whole tree and fruit features of Changlin40. Figure 2 Estimation of Changlin40 genome size by 17‐mers depth distribution of 519 Gb raw Illumina sequence data. Two peaks are observed (49× and 104×): the first peak represents the unique part of the genome and the second peak represents the repetitive part of the genome. Figure 3 Meiosis process of the pollen mother cells in Changlin40. Figure 4 Hi‐C heatmap showing the chromosomal interactions of intra‐ and inter‐chromosomal within Changlin40 genome. Figure 5 Synteny of the Changlin40 genome comparison with Camellia oleifera (Lin et al., 2022) and Camellia lanceoleosa (Gong et al., 2022). Figure 6 The distribution of structural variation count between the hexaploid Changlin40 and diploid. Figure 7 KEGG pathways enrichment analysis of Changlin40‐specific genes. The color of circle represents the FDR (false discovery rate) in the hypergeometric test corrected using BH (Benjamini and Hochberg) method. The size of circle represents the gene count of the KEGG terms. Figure 8 KEGG pathways enrichment analysis of Changlin40 expansion genes. The color of circle represents the FDR (false discovery rate) in the hypergeometric test corrected using BH (Benjamini and Hochberg) method. The size of circle represents the gene count of the KEGG terms. Figure 9 Barplot showing co‐expression modules size identified by weighted correlation network analysis (WGCNA) across seed kernel development stages in Changlin40. Figure 10 Statistics pertaining to the contents of primary unsaturated and saturated fatty acids in Camellia oil during the developmental stages of Changlin40 seed kernels at relative (A) and absolute (B) levels. Figure 11 Analysis of gene involved in Camellia oil biosynthesis. Figure 12 The statistical distribution of structural gene and transcription factors contained in the lipid metabolism regulat [file PBI-22-2890-s001.zip › Supplementary information.pdf]

The complex hexaploid oil-Camellia genome traces back its phylogenomic history and multi-omics analysis of Camellia oil biosynthesis

Table of Contents

Table of Contents 2

1. Supplementary Note 3

    1.1 Classification of glyceride metabolites from the seed kernel of Changlin40 3

2. Supplementary Figure 4

3. Supplementary Table 15

1. Supplementary Note

1.1 Classification of glyceride metabolites from the seed kernel of Changlin40

A total of 349 glyceride metabolites were identified, which can be categorized into 13 distinct lipid types, inclusive diacylglycerol (DAG) with 18 subclasses, triacylglycerol (TAG) with 248 subclasses, monogalactosyldiacylglycerol (MGDG) with 5 subclasses, digalactosyldiacylglycerol (DGDG) with 9 subclasses, sulfoquinovosyl diacylglycerol (SQDG) with 4 subclasses, phosphatidic acid (PA) with 10 subclasses, phosphatidylcholine (PC) with 15 subclasses, phosphatidyl ethanolamine (PE) with 13 subclasses, phosphatidylglycerol (PG) with 6 subclasses, phosphatidylinositol (PI) with 10 subclasses, lysophosphatidylcholine (LPC) with 5 subclasses, lysophosphatidyl ethanolamine (LPE) with 4 subclasses and lysophosphatidylglycerol (LPG) with 2 subclasses (Figure 6B and Supplementary Table 9).

2. Supplementary Figure

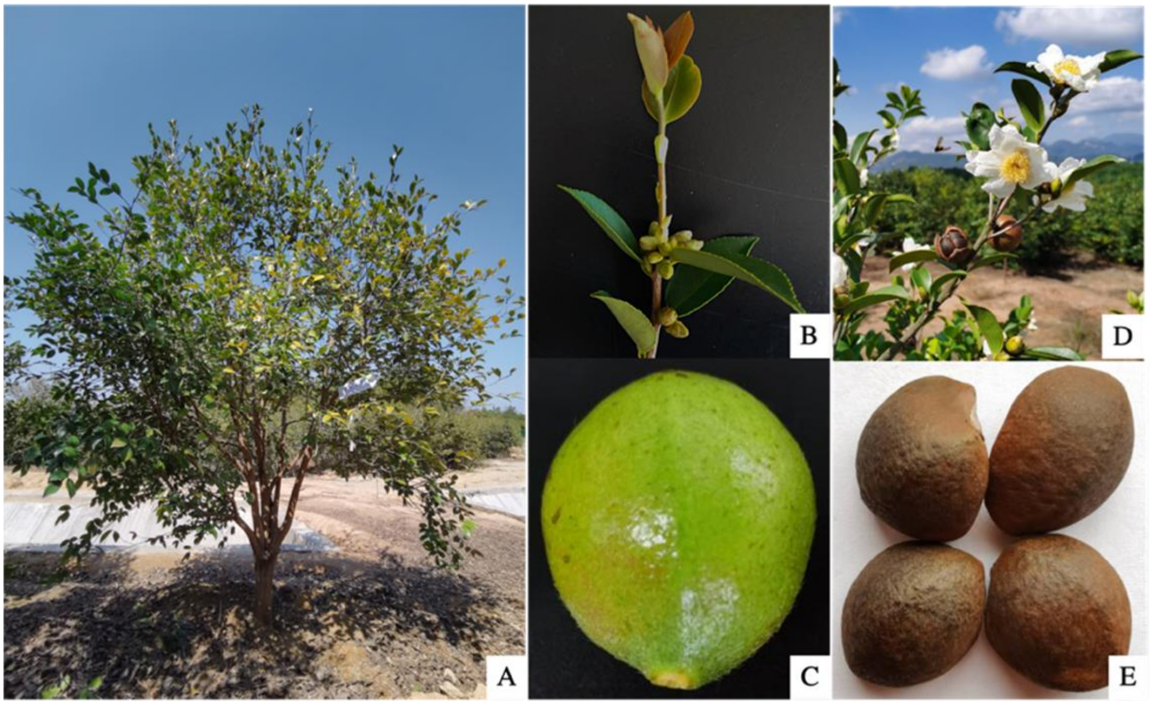

Supplementary Figure 1. The whole tree and fruit features of Changlin40. (A) The whole tree of Changlin 40. (B) Floral buds and young branches of Changlin40. (C) Fruits of Changlin40. (D) Flowers and mature tea fruits of Changlin40. (E) Seeds of Changlin40.

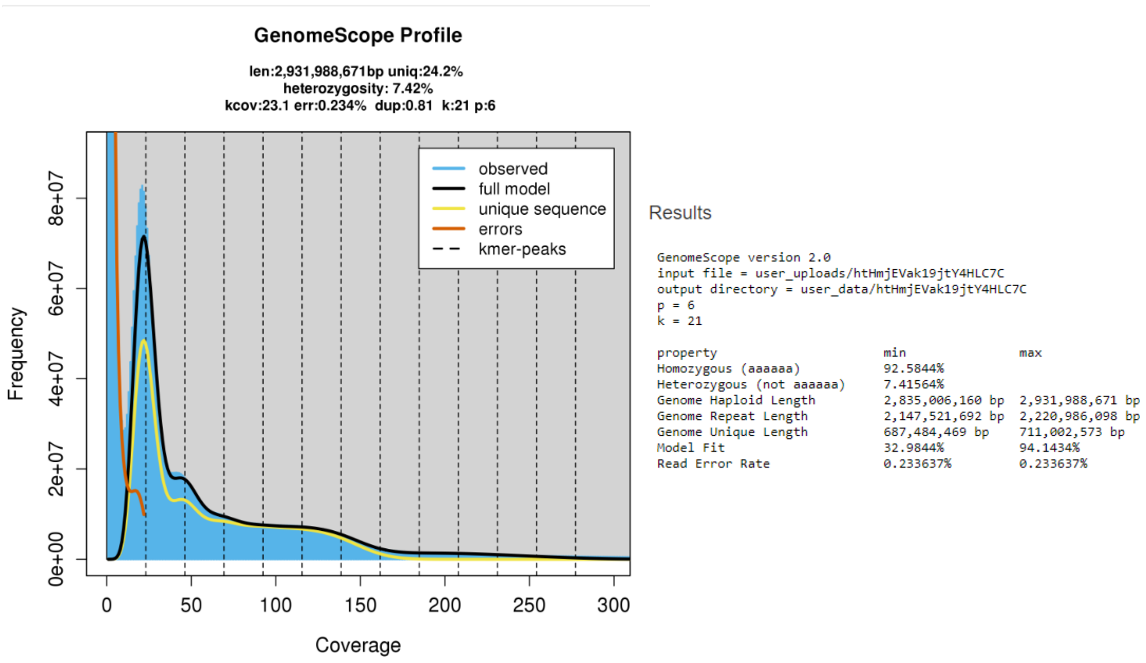

Supplementary Figure 2. Estimation of Changlin40 genome size by 17-mers depth distribution of 519 Gb raw Illumina sequence data. Two peaks are observed (49× and 104×): the first peak represents the unique part of the genome and the second peak represents the repetitive part of the genome.

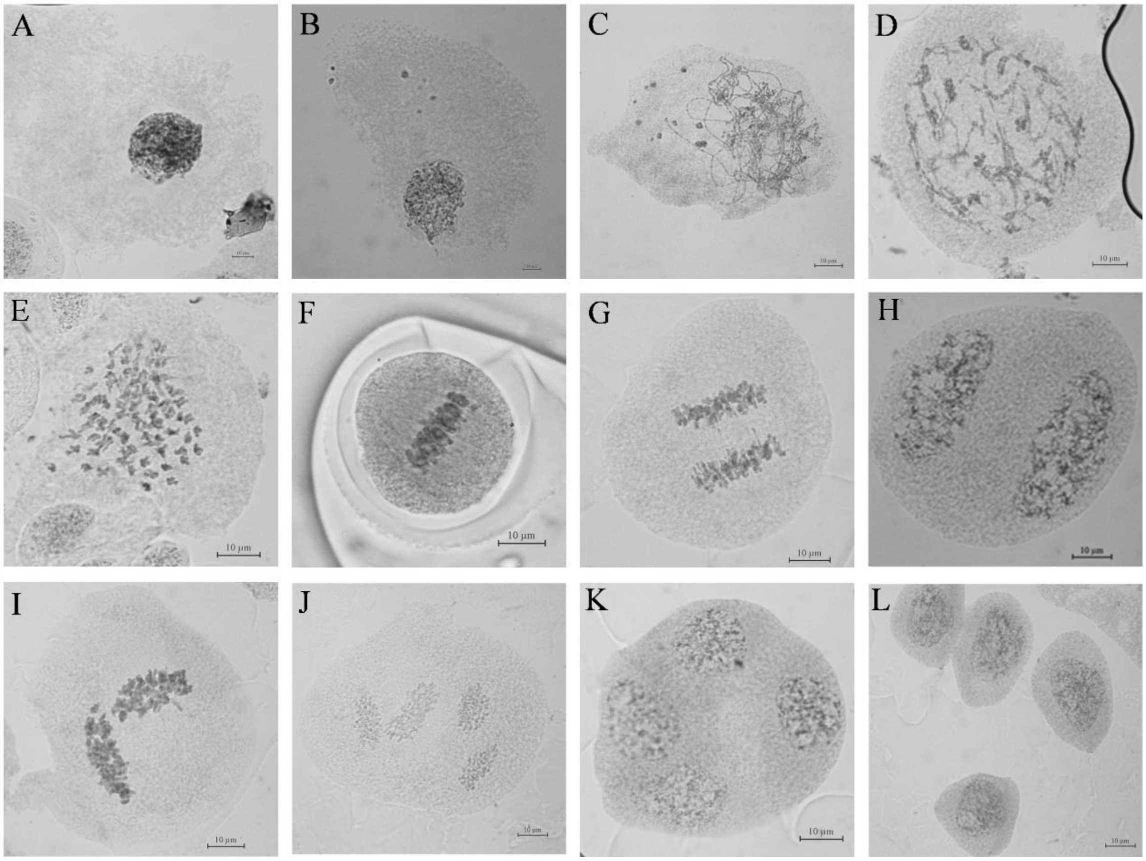

Supplementary Figure 3. Meiosis process of the pollen mother cells in Changlin40. A-M. Pollen mother cells at different meiotic stage. A. Leptotene; B. Zygotene; C. Pachytene; D. Diplotene; E. Diakinesis; F. Metaphase I; G. Anaphase I; H. Prophase II; I. Metaphase II; J. Anaphase II; K. Telophase II; L. Tetrad. Scale bar: 10 μM.

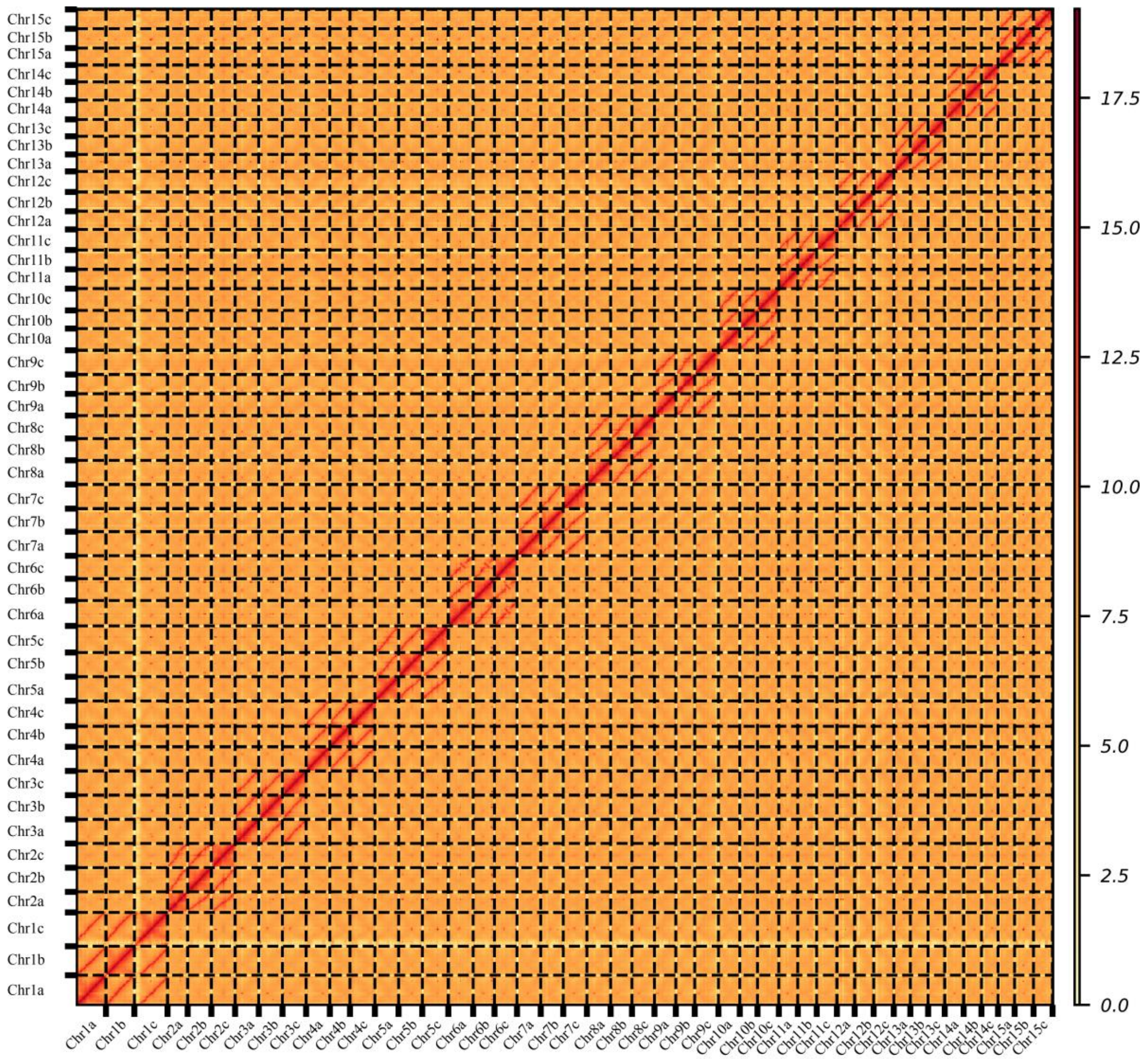

Supplementary Figure 4. Hi-C heatmap showing the chromosomal interactions of intra- and inter-chromosomal within Changlin40 genome.

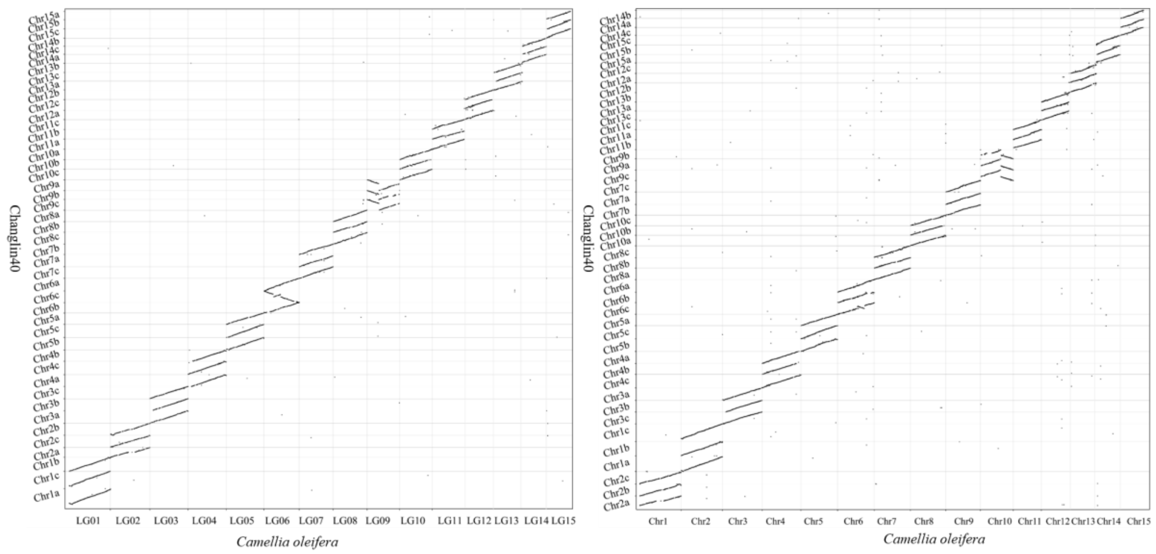

Supplementary Figure 5. Synteny of the Changlin40 genome comparison with *Camellia oleifera* (Lin et al., 2022) and *Camellia lanceoleosa* (Gong et al., 2022).

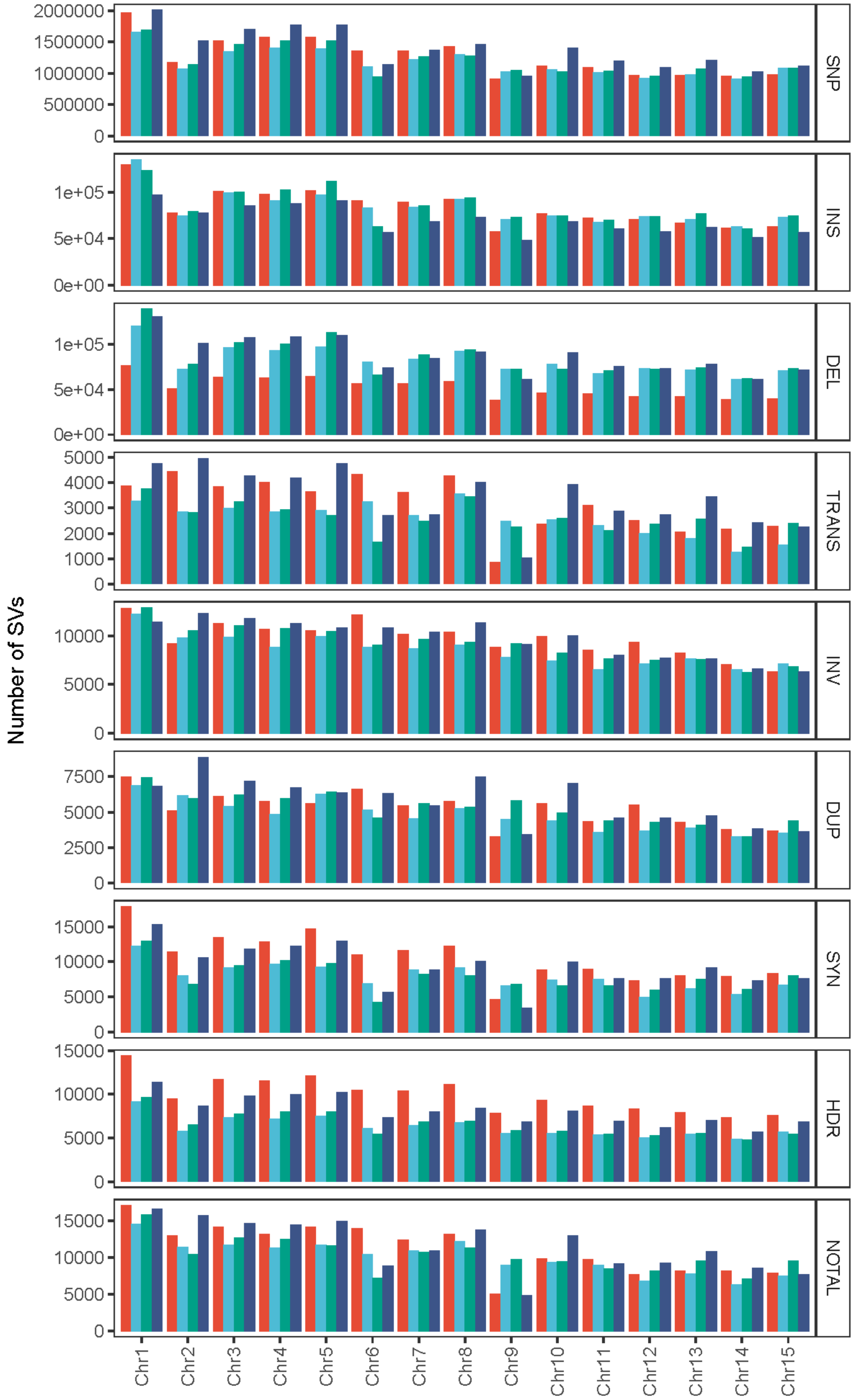

Supplementary Figure 6. The distribution of structural variation count between the hexaploid Changlin40 and diploid.

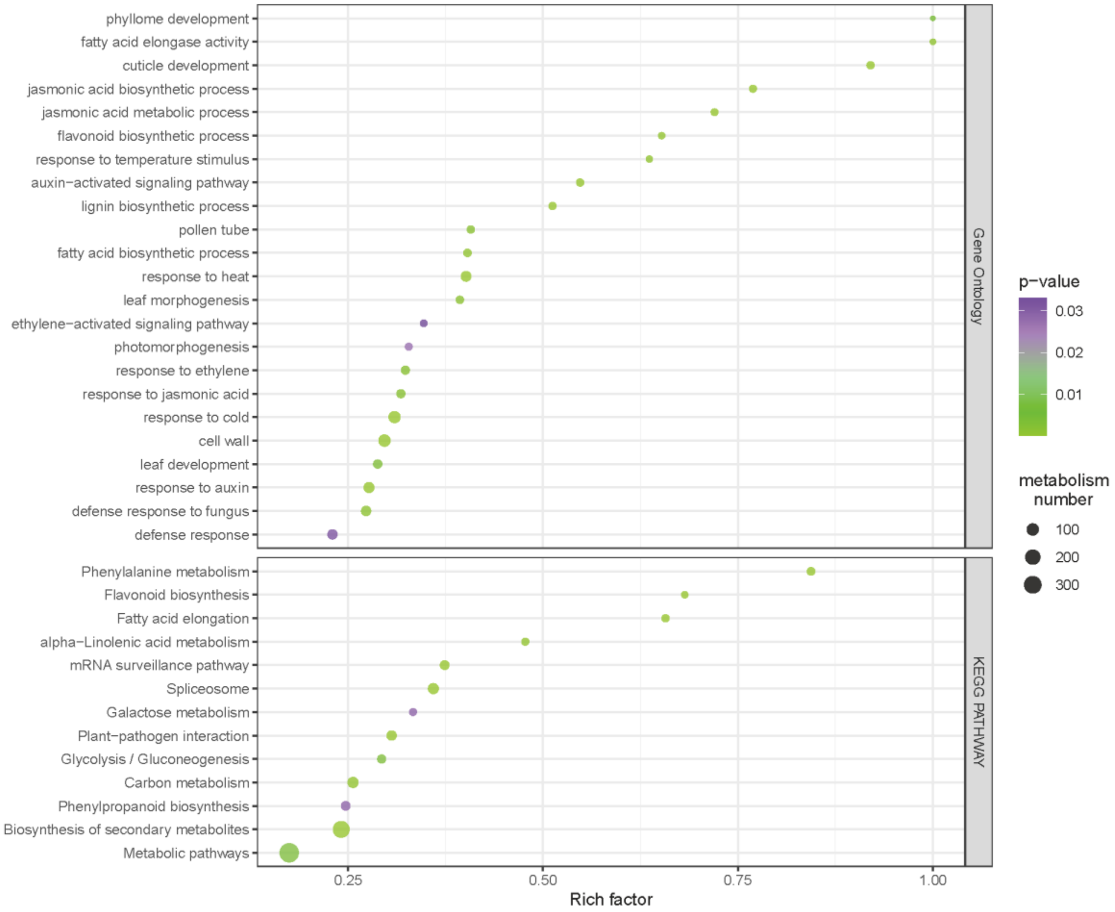

Supplementary Figure 7. KEGG pathways enrichment analysis of Changlin40-specific genes. The color of circle represents the FDR (false discovery rate) in the hypergeometric test corrected using BH (Benjamini and Hochberg) method. The size of circle represents the gene count of the KEGG terms.

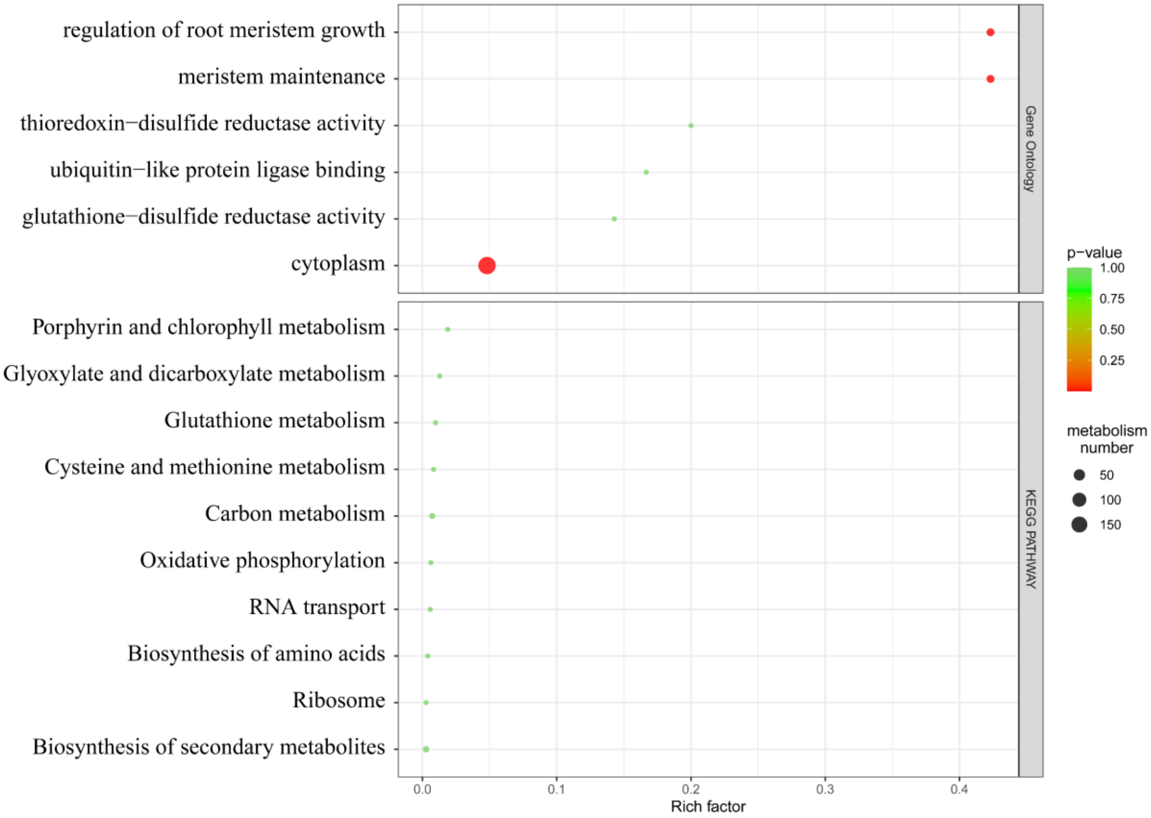

Supplementary Figure 8. KEGG pathways enrichment analysis of Changlin40 expansion genes. The color of circle represents the FDR (false discovery rate) in the hypergeometric test corrected using BH (Benjamini and Hochberg) method. The size of circle represents the gene count of the KEGG terms.

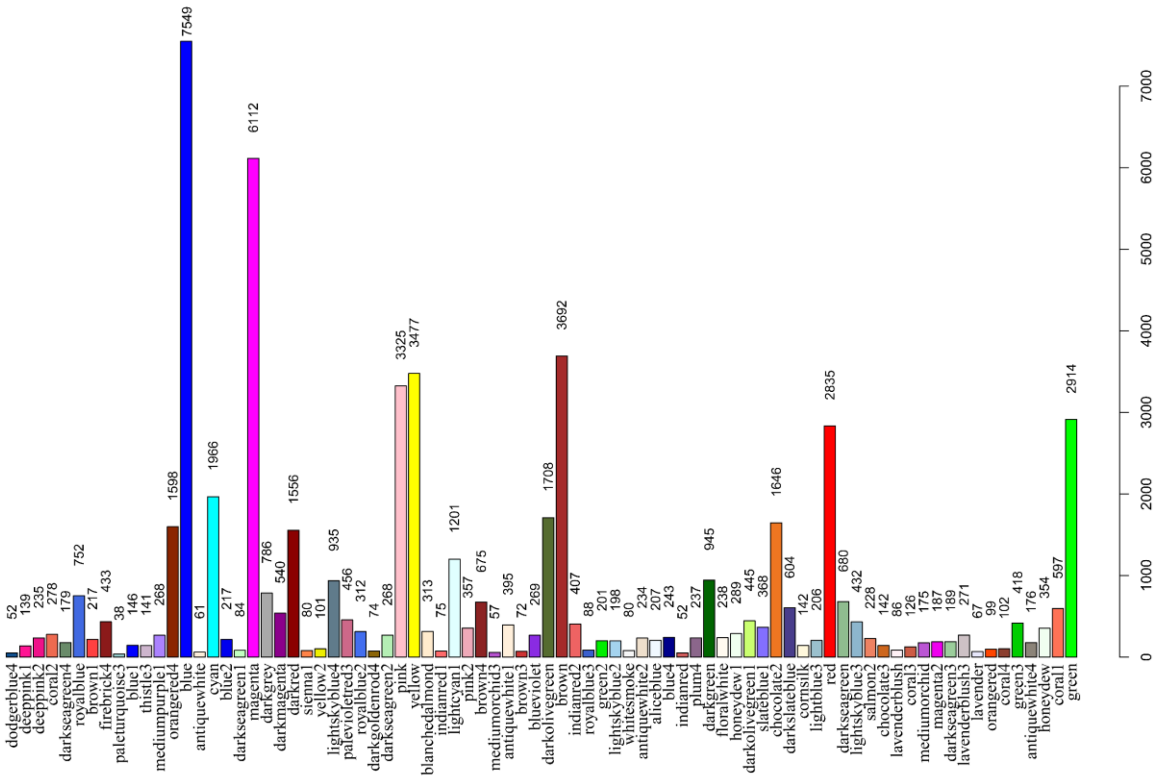

Supplementary Figure 9. Barplot showing co-expression modules size identified by weighted correlation network analysis (WGCNA) across seed kernel development stages in Changlin40.

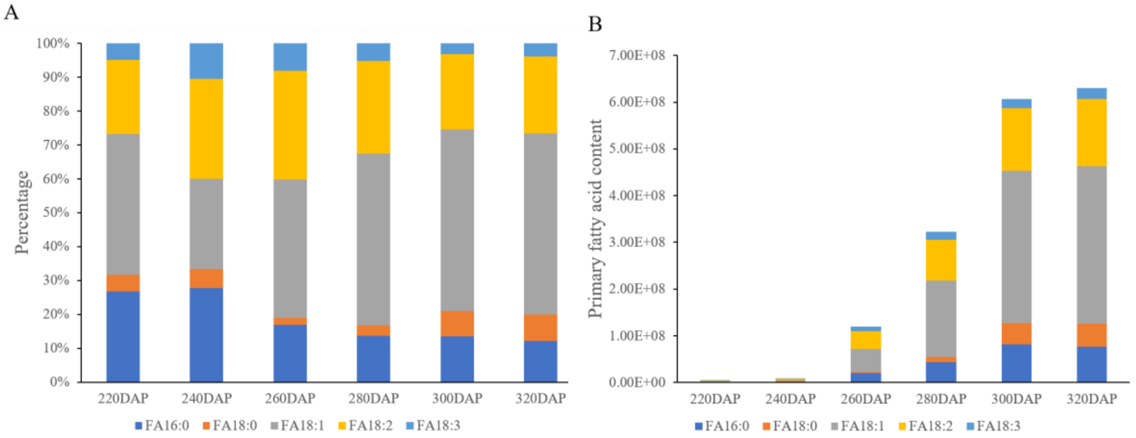

Supplementary Figure 10. Statistics pertaining to the contents of primary unsaturated and saturated fatty acids in Camellia oil during the developmental stages of Changlin40 seed kernels at relative (A) and absolute (B) levels.

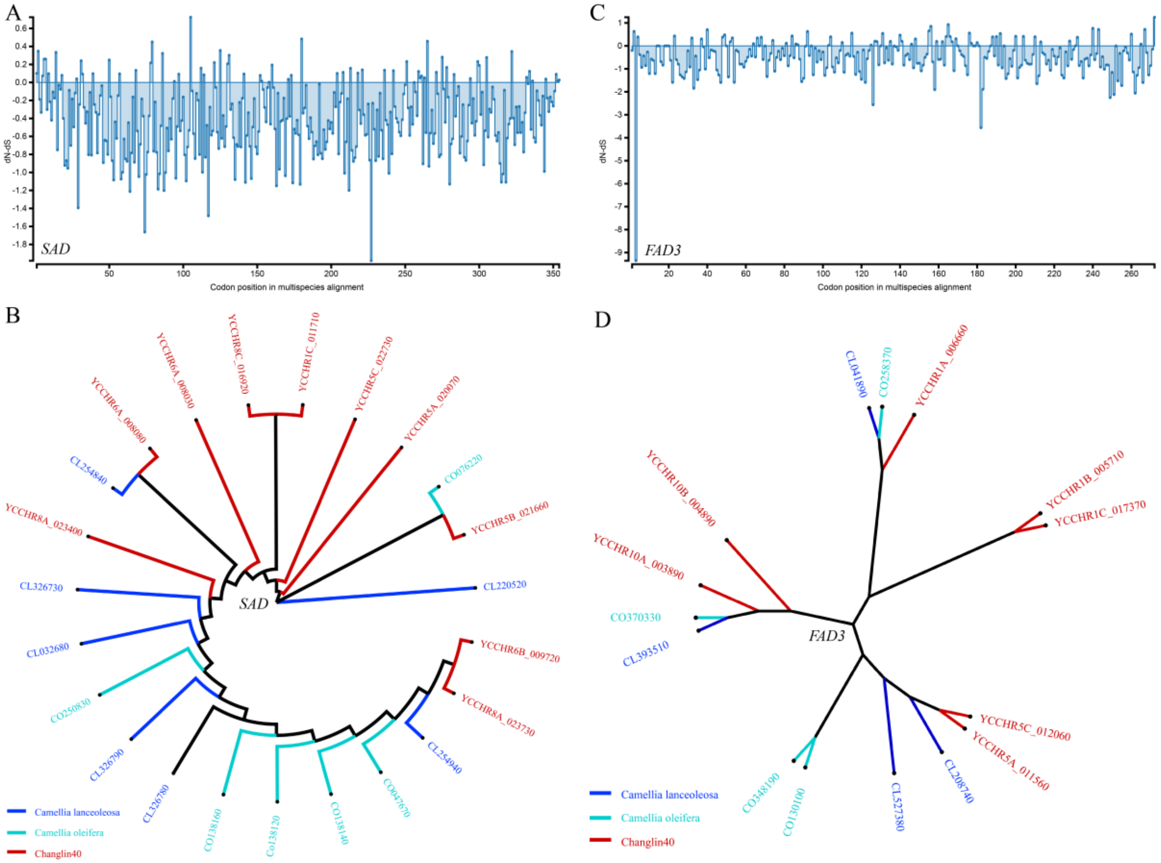

Supplementary Figure 11. Analysis of gene involved in Camellia oil biosynthesis. dN-dS values across a multispecies alignment of *SAD* (A) and *FAD3* (C) gene sequences. Points indicate statistically significant evidence for codons under selection. Phylogenetic tree of *SAD* (B) and *FAD3* (D) gene using the ML method.

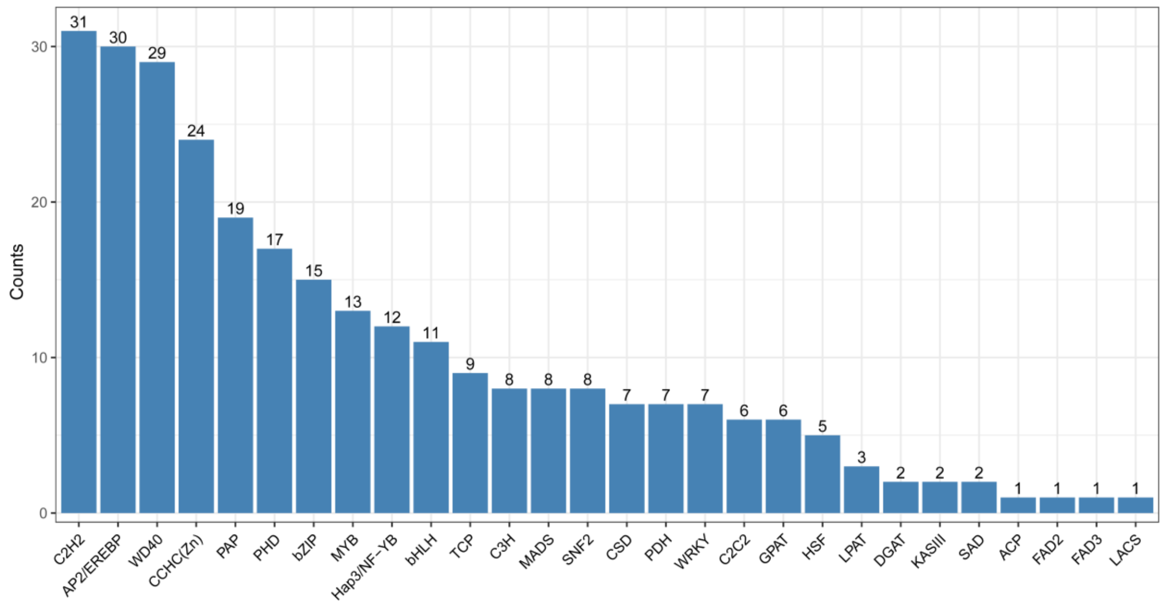

Supplementary Figure 12. The statistical distribution of structural gene and transcription factors contained in the lipid metabolism regulation network of seed kernel development in Changlin40.

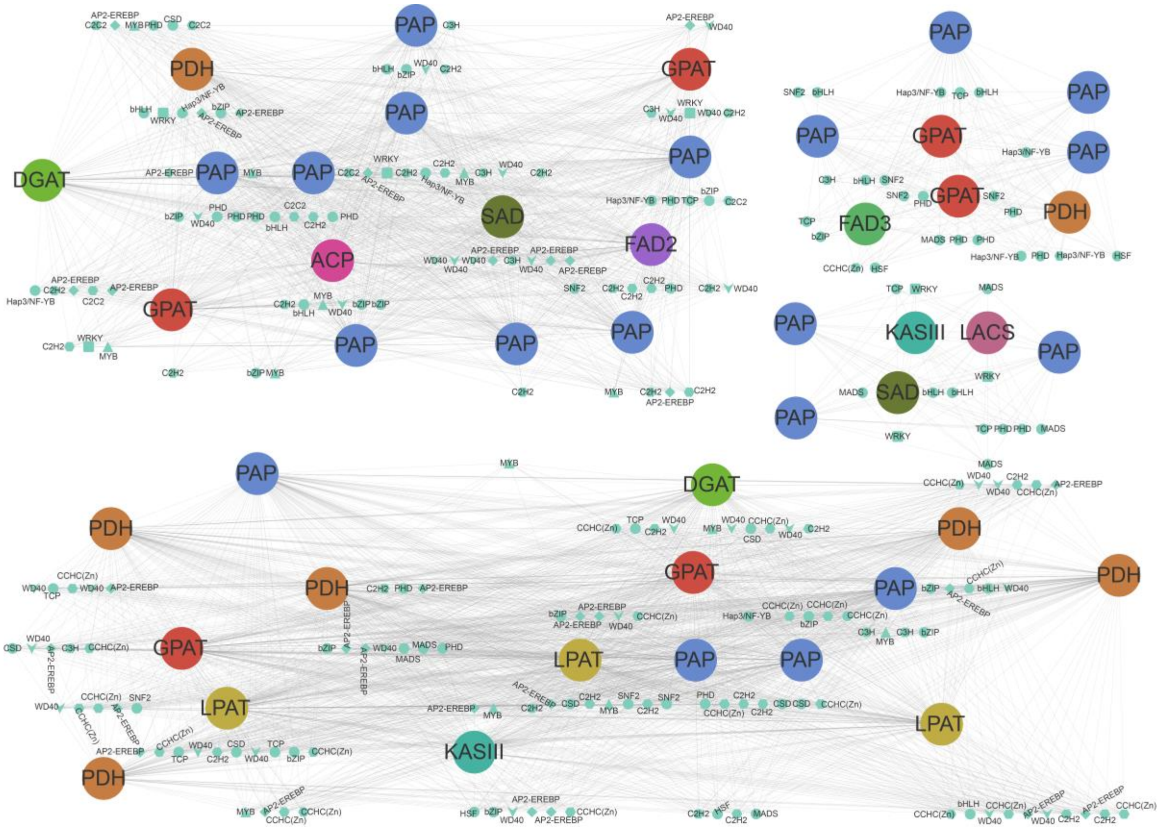

Supplementary Figure 13. Network built based on the correlation between genes related to lipid metabolites. The structural genes associated with lipid metabolism were depicted as colored dots, while the TFs were represented by small cyan graphs. All modules significantly correlated with oil-related metabolites were shown in this network.

3. Supplementary Table

Supplementary Table 1: Summary of Illumina reads (DNA) for oil-Camellia.

Supplementary Table 2: Summary of PacBio HiFi reads for oil-Camellia.

Supplementary Table 3: Summary of HiC reads for oil-Camellia.

Supplementary Table 4: Summary of oil-Camellia genome assembly.

Supplementary Table 5: The telomere sequence in fragmentary scaffolds that were not anchored to chromosomes.

Supplementary Table 6: Summary of repeats in oil-Camellia genome.

Supplementary Table 7: Summary of RNA-seq and Iso-seq reads of oil-Camellia.

Supplementary Table 8: General statistics of predicted protein-coding genes.

Supplementary Table 9: The lipid concentrations of *Camellia oleifera* seed kernels in six different development stages.

Supplementary Table 10: Mean and standard deviation of total lipid concentrations of *Camellia oleifera* seed kernels in six different development stages.

Supplementary Table 11: The independent t-test of samples from *Camellia oleifera* seed kernels in six different development stages.
